# Anticholinergic Burden and Progression to Kidney Replacement Therapy

**DOI:** 10.1016/j.ekir.2026.106552

**Published:** 2026-04-20

**Authors:** Agathe Mouheb, Ziad A. Massy, Marie Metzger, Hélène Levassort, Marion Pépin, Maurice Laville, Natalia Alencar de Pinho, Solène M. Laville, Sophie Liabeuf, Chronic Kidney Disease–Renal Epidemiology and Information Network (CKD-REIN) Study Group, Natalia Alencar de Pinho, Dorothée Cannet, Christian Combe, Denis Fouque, Luc Frimat, Aghilès Hamroun, Yves-Edouard Herpe, Christian Jacquelinet, Oriane Lambert, Céline Lange, Maurice Laville, Sophie Liabeuf, Ziad A. Massy, Marie Metzger, Pascal Morel, Christophe Pascal, Roberto Pecoits-Filho, Joost Schantsra, Bénédicte Stengel, T. Hannedouche, B. Moulin, A. Klein, C. Combe, J.P. Bourdenx, A. Keller, C. Delclaux, B. Vendrely, B. Deroure, A. Lacraz, T. Lobbedez, I. Landru, Z. Massy, P. Lang, X. Belenfant, E. Thervet, P. Urena, M. Delahousse, C. Vela, M. Essig, D. Clément, H. Sekhri, M. Smati, M. Jamali, B. Hacq, V. Panescu, M. Bellou, Luc Frimat, N. Kamar, C. Noël, F. Glowacki, N. Maisonneuve, R. Azar, M. Hoffmann, M. Hourmant, A. Testa, D. Besnier, G. Choukroun, G. Lambrey, S. Burtey, G. Lebrun, E. Magnant, M. Laville, D. Fouque, L. Juillard, C. Chazot, P. Zaoui, F. Kuentz

**Affiliations:** 1 Pharmacoepidemiology Unit, Department of Clinical Pharmacology, Amiens-Picardie University Medical Center, Amiens, France; 2 MP3CV Laboratory, Jules Verne University of Picardie, Amiens, France; 3 Centre for Research in Epidemiology and Population Health (CESP), INSERM UMRS 1018, Université Versailles Saint Quentin, Université Paris-Saclay, Villejuif, France; 4 Department of Nephrology, Ambroise Paré University Hospital, APHP, Paris, France; 5 AURA Paris, Paris, France; 6 Geriatric Department, University Medical Department 1, AP-HP, Ambroise Paré Hospital, University Hospital Group Paris Saclay, Boulogne-Billancourt, France; 7 CarMeN INSERM 1060, Université de Lyon, Lyon, France

**Keywords:** anticholinergic, chronic kidney disease, drug use, kidney replacement therapy, pharmacoepidemiology

## Abstract

**Introduction:**

The cholinergic system exerts nephroprotective effects through antiinflammatory and vasoregulatory pathways. However, the impact of anticholinergic medications on chronic kidney disease (CKD) progression remains unexplored. This study aimed to evaluate the association between anticholinergic burden and CKD progression to kidney replacement therapy (KRT).

**Methods:**

Nephrology outpatients with a confirmed diagnosis of CKD (estimate glomerular filtration rate [eGFR] < 60 ml/min per 1.73 m^2^) were enrolled in the Chronic Kidney Disease – Renal Epidemiology and Information Network (CKD-REIN) prospective cohort study. CKD progression (defined as the initiation of KRT) were recorded prospectively during the 5-year follow-up period. For each patient, anticholinergic burden was determined by summing the Anticholinergic Cognitive Burden (ACB) scores of all prescription drugs at baseline. We used multivariable, cause-specific, Cox proportional hazards regression models to analyze the association between anticholinergic burden and CKD progression.

**Results:**

Of the 3009 patients (median age at baseline: 69 years; men: 66%; mean eGFR: 34 ml/min per 1.73 m^2^), 647 initiated KRT and 405 died during a median follow-up period of 5 years. A high anticholinergic burden was independently associated with CKD progression (hazard ratio [HR] = 1.53, 95% confidence interval [CI] = 1.09–2.14), relative to a null burden. A low or moderate anticholinergic burden was associated with an elevated risk (HR [95% CI] = 1.09 [0.85–1.24]), although not significantly.

**Conclusion:**

A high anticholinergic burden was associated with a greater risk of CKD progression. This finding suggests that the disruption of cholinergic signaling could contribute to a decline in renal function and highlights the importance of medication review and medication optimization in CKD management.

## Graphical abstract

**Figure:**
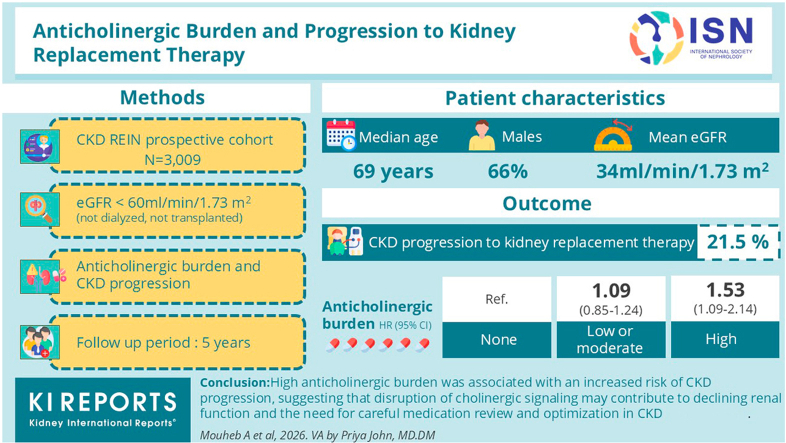

CKD affects approximately 10% to 15% of the global adult population. Given the natural progression of the disease, CKD poses a major public health burden.^1^^,^^2^ Preventing progression to KRT by dialysis or transplantation remains a crucial health care goal, because this progression results in significant morbidity, mortality, and societal costs.^1^^,^^2^

Understanding modifiable factors that may influence CKD progression is a major part of the clinical management of CKD. Along with conventional risk factors, a growing body of evidence has highlighted the role of the cholinergic system in maintaining kidney homeostasis and modulating kidney blood flow, inflammation, and tubular function.^3^, ^4^, ^5^ The cholinergic antiinflammatory pathway is mediated by the vagus nerve and acetylcholine, and stimulation of this pathways has been shown to protect against kidney injury in animal models of CKD.^6^, ^7^, ^8^, ^9^, ^10^, ^11^, ^12^, ^13^, ^14^, ^15^ In a study of a cohort patients with Alzheimer’s dementia, the use of cholinesterase inhibitors (which increase acetylcholine levels) was associated with slower CKD progression (HR [95% CI]: 0.82 [0.71–0.96]), compared with nonuse.^16^ Given the intricate interplay between kidney function and the cholinergic system, interventions targeting the latter might offer a novel treatment approach for CKD. However, potential interventions have not been investigated extensively. A pilot interventional study in dialysis patients explored the therapeutic potential of cholinergic stimulation but did not find any significant changes in levels of proinflammatory cytokine.^17^

Although cholinergic stimulation may confer kidney benefits, many patients with CKD are exposed to medications with anticholinergic properties.^18^ These agents antagonize cholinergic receptors and might therefore disrupt protective mechanisms and accelerate kidney damage. Moreover, in the general population and in older patients, a higher anticholinergic burden has been linked to adverse outcomes, including functional decline, hospitalization, and mortality.^19^, ^20^, ^21^ Whether similar associations exist in patients with CKD, particularly regarding kidney disease progression and mortality, remains unknown. To date, the potential impact between the anticholinergic burden from prescription drugs taken by patients with CKD on progression of their renal disease or mortality has not yet been investigated specifically. We hypothesized that a high anticholinergic burden is associated with CKD progression and increased mortality.

To the best of our knowledge, the putative association between the anticholinergic burden and renal outcomes in nondialyzed patients with CKD has not previously been examined. Therefore, the objective of the present study of patients with moderate-to-severe CKD (eGFR < 60 ml/min per 1.73 m^2^) being managed by a nephrologist in France was to analyze the putative association between the anticholinergic burden and CKD progression (defined as the initiation of KRT) and all-cause mortality.

## Methods

### Study Design and Participants

The prospective CKD-REIN cohort study was conducted in 40 nationally representative nephrology outpatient clinics in France. The main inclusion criteria were age ≥ 18 years, management by a nephrologist, a diagnosis of stage 3 or 4 CKD, and no history of KRT (i.e., no previous maintenance dialysis or kidney transplantation). Details of the study protocol have been published elsewhere.^22^ A total of 3033 patients were included between July 2013 and April 2016 and were actively followed-up with by trained clinical research associates for 5 years. The study protocol was approved by the institutional review board at the French National Institute of Health and Medical Research (INSERM; reference: IRB00003888) and registered at ClinicalTrials.gov (NCT03381950).

### Study Data

Using interviews, medical records, and patient self-questionnaires, trained clinical research associates collected data at baseline and then at 1-year intervals. The variables included patient characteristics (age, sex, educational level, smoking status, and body mass index) and comorbidities (past and/or present cardiovascular disease, systolic blood pressure, diabetes mellitus, and autosomal dominant polycystic kidney disease). Variables were defined in Supplementary Table S1. All patients were prescribed a set of standard blood and urine tests (as recommended by the French health authorities for routine CKD care) to be performed at their usual medical laboratory. This set included assays of serum creatinine, urea, uric acid levels, and calculation of the urinary albumin-to-creatinine ratio.

Patients were asked to bring all their drug prescriptions (regardless of the prescribing physician) for the 3 months immediately preceding the interview with a clinical research associate and for each year of follow-up. Over-the-counter medications were not documented. The clinical research associate used an electronic case report form (linked to the international Anatomical Therapeutic and Chemical thesaurus) to enter standardized Anatomical Therapeutic and Chemical codes. The period of prescription, trade name, Anatomical Therapeutic and Chemical class, unit dose, daily dose, and pharmaceutical formulation were reported for each drug prescription.

### Anticholinergic Burden

We used the ACB scale to estimate the anticholinergic burden^23^; this is the tool most commonly used to measure the anticholinergic burden in the context of cognitive impairment (a central adverse event) and has been ranked highest in a systematic quality assessment.^24^ This scale has been validated in older populations and is based on expert consensus, serum anticholinergic activity, or muscarinic receptor affinity.^25^ The anticholinergic burden for each patient at baseline was determined by summing the ACB scores for all prescription drugs with anticholinergic properties. If a study participant had not been prescribed any prescription drugs with anticholinergic properties, the anticholinergic burden was considered to be null. Each individual’s anticholinergic burden was classified as null (ACB score = 0), low (ACB score = 1), moderate (ACB score = 2), or high (ACB score ≥ 3). For the purposes of our analysis, we further categorized anticholinergic burden into 3 groups, namely, null, low or moderate, and high, in order to evaluate its specific impact.

We performed a sensitivity analysis by assessing the Anticholinergic Risk Scale (ARS) score, an index of central and peripheral nervous system adverse drug reactions developed in a population of older men.^26^ Although the ACB scale and the ARS both measure the anticholinergic burden, they were built in different ways.^25^ As a result of these differences, the ARS identified fewer drugs (49 in total and 21 with a score of 3) than the ACB scale did (84 in total and 38 with a score of 3); however, we reasoned that convergent findings with 2 independent scales would strengthen our results. The principles described above for the ACB scale were also applied to summing and interpretation of the ARS score.

### Study Outcomes

Medical events were recorded prospectively throughout the active follow-up period. The primary outcome was CKD progression, defined as the initiation of KRT (i.e., maintenance dialysis or preemptive kidney transplantation) documented in medical records, patient interviews, and/or through linkage to the French National Renal Epidemiology and Information Network registry.^27^

Deaths were ascertained from death certificates, hospital records, reports by family members, and linkage to the national vital status registry. Patients were censored at the date of the competing event, the end of their 5-year follow-up period, or at loss to follow-up. Death before KRT initiation was considered a secondary outcome.

### Statistical Analyses

The characteristics of patients in the CKD-REIN cohort at baseline were described overall and for anticholinergic burden subgroups (no, low or moderate, or high anticholinergic burdens, as defined above) and were compared using standardized mean differences. Categorical variables were reported as the frequency (percentage). Continuous variables were reported as the mean ± SD or the median (interquartile range), depending on the distribution of the data.

The most frequently prescribed Anatomical Therapeutic and Chemical codes in patients with a high anticholinergic burden were reported as a percentage. The medications or combinations of prescribed medications in this group were presented graphically.

Incidence rates (IRs) and the corresponding 95% CIs were calculated for KRT initiation and death. Cause-specific Cox proportional hazards models were used to examine the association between the anticholinergic burden and the incidences of KRT initiation and death. Each Cox model was adjusted for a predefined set of confounders identified using a direct acyclic graph, a visual representation of the assumed causal relationships among variables. This approach allows for the selection of the minimal sufficient adjustment sets, that is, covariates associated with both the exposure and the outcome, while avoiding overadjustment.^28^^,^^29^ For the model assessing KRT initiation, the adjustment set included age, sex, history of cardiovascular disease, diabetes mellitus, dyslipidemia, systolic blood pressure, autosomal dominant polycystic kidney disease, number of daily drugs, and compliance. Given the nature of our study, eGFR was additionally included (Supplementary Figure S1). For the mortality model, the adjustment set included age, sex, educational level (years), smoking status, eGFR, urea, uric acid, body mass index, systolic blood pressure, history of cardiovascular disease, use of antihypertensive drugs and lipid modifying agents, number of daily drugs, and compliance (Supplementary Figure S2). The proportional hazards assumption was assessed by examining the Schoenfeld residuals. HRs were presented with their corresponding 95% CIs. Potential modification of the effect of the anticholinergic burden by age, sex, and eGFR was assessed by including an interaction term in the models (*P* for interaction < 0.05 was considered to be statistically significant). When appropriate, stratified analyses were then performed.

To test the robustness of our results, we performed several sensitivity analyses. First, all the analyses were repeated by using the ARS as an alternative measure of the anticholinergic burden. Second, and given that higher levels of albuminuria are strongly associated with CKD progression and mortality, we further adjusted our models for the urinary albumin-to-creatinine ratio even though this variable was not part of the minimal sufficient adjustment set identified in the direct acyclic graph. Lastly, to account for the competing risk of death, we fitted a Fine and Gray subdistribution hazard model (in the adjusted curves R package^30^).

For missing data, we used multiple imputation by chained equations with 20 iterations and the following: (i) 10 datasets for the model with KRT as the outcome or (ii) 16 datasets for the model with death as the outcome. The imputation models included all the covariates used in the adjusted analyses. A separate imputation model was built for each analysis. The number of imputed datasets was determined using White *et al.*'s equation^31^ as m = 100 − percentage of complete cases. This number depended on the model, given the differences in the included variables.

The results of this cohort study are reported in accordance with the Strengthening the Reporting of Observational Studies in Epidemiology guidelines.^32^ The threshold for statistical significance was set to *P* < 0.05. Statistical analysis was performed using R software (version 4.3.0).^33^

## Results

Among the 3033 patients in the CKD-REIN cohort, 11 had no follow-up data, and 13 had no prescription data. Thus, 3009 patients were included in the present analysis.

### Baseline Characteristics

**Figure 1:**
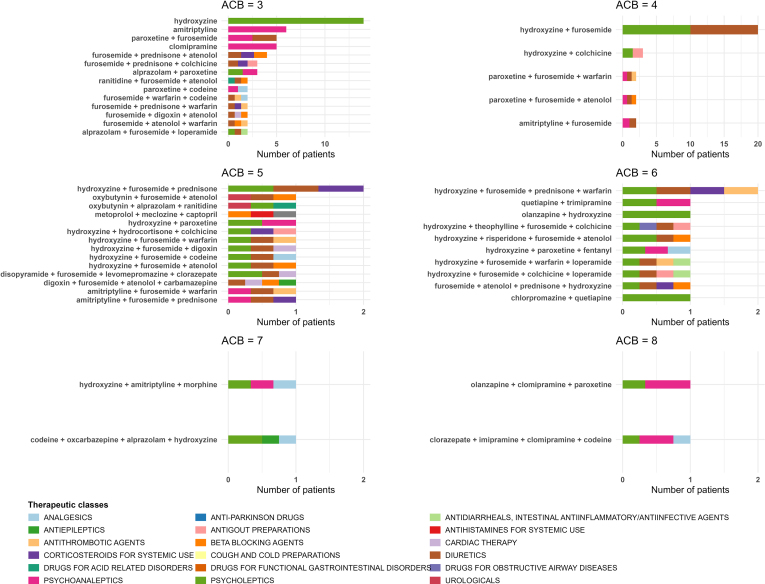
Medications or combinations of medications leading to a high anticholinergic burden (ACB score ≥ 3). ACB, anticholinergic cognitive burden.

At baseline, the median (interquartile range) age was 69 (60–76) years, and majority of the participants were men (66%). The mean ± SD eGFR was 34 ± 13 ml/min per 1.73 m^2^, and the mean ± SD systolic blood pressure was 142 ± 20 mm Hg. Majority of the participants had a history of cardiovascular disease (53%), and/or dyslipidemia (74%). At baseline, 1556 (52%) patients were taking ≥ 1 prescription drug with anticholinergic properties. Of these, 1393 (90%) had a low (1099 [71%]) or moderate (294 [19%]) anticholinergic burden, and 163 (10%) had a high anticholinergic burden (Table 1). Many of these patients (58%) were taking a diuretic with an ACB score of 1, 37% were taking a psycholeptic with an ACB score of 3, 16% were taking a psychoanaleptic with an ACB score of 2, and 15% were taking a beta-blocker with an ACB score of 1 (Table 2). When we focused on prescriptions resulting in a high anticholinergic burden, we observed that majority of patients were taking furosemide in combination with hydroxyzine or another drug (Figure 1, Supplementary Table S2).

**Table 1:** Baseline characteristics of the patients in the CKD-REIN cohort, as a function of the anticholinergic burden (calculated using the ACB score)

Characteristics	All (*N* = 3009)	Anticholinergic burden			SMD	Imputed data (%)
		Null (*n* = 1453)	Low or moderate (*n* = 1393)	High (*n* = 163)		
Sociodemographic factors						
Age (yrs)	69 (60–76)	67 (57–74)	70 (63–78)	70 (62–77)	0.27	-
Men	65.5%	65.8%	66.8%	52.1%	0.20	-
Educational level					0.37	1.2%
< 9 yrs	14.8%	11.3%	17.9%	19.2%		
9–11 yrs	49.2%	45.1%	52.1%	61.2%		
≥ 12 yrs	36.0%	43.6%	30.0%	19.5%		
Smoking status					0.12	0.8%
Current smoker	11.8%	13.4%	10.2%	11.1%		
Exsmoker	47.0%	44.7%	49.9%	43.1%		
Nonsmoker	41.2%	41.9%	40.0%	45.9%		
Clinical and laboratory variables						
BMI (kg/m^2^)	28.7 ± 5.8	27.4 ± 5.2	29.9 ± 6.1	30.6 ± 6.5	0.36	2.1%
Obesity (BMI ≥ 30 kg/m^2^)	35.5%	26.3%	43.8%	47.9%		
Systolic blood pressure (mmHg)	141.5 ± 19.7	140 ± 19.1	143 ± 20.2	142.3 ± 20.4	0.10	0.6%
eGFR (ml/min per 1.73 m^2^)	34.0 ± 13.0	36.3 ± 13.3	31.8 ± 12.5	32.4 ± 11.4	0.24	-
Urinary albumin-to-creatinine ratio (mg/g)					0.19	11.9%
A1 (< 30 mg/g)	30.2%	31.9%	29.1%	24.2%		
A2 (30–300 mg/g)	35.3%	35.6%	35.5%	31.0%		
A3 (≥ 300 mg/g)	34.5%	32.5%	35.5%	44.8%		
Urea (mmol/l)	12.8 (9.7–17.1)	11.5 (8.8–15.1)	14.3 (10.7–19.2)	13.9 (10.7–19.6)	0.38	4.2%
Phosphate (mg/dl)	1.2 ± 0.2	1.1 ± 0.2	1.2 ± 0.2	1.2 ± 0.2	0.15	3.9%
Uric acid (μmol/l)	430.6 ± 120	411.4 ± 107.9	448 ± 127.4	452.7 ± 130.6	0.23	8.2%
Medical history						
Cardiovascular disease	53.1%	39.8%	66.0%	61.0%	0.36	1.3%
Diabetes mellitus	43.4%	31.6%	53.8%	60.7%	0.41	0.2%
Dyslipidemia	73.7%	66.9%	80.0%	79.9%	0.20	0.2%
ADPKD	5.8%	7.9%	3.9%	2.6%	0.16	5.9%
Medication use						
Antihypertensive agent	93.2%	89.3%	97.1%	95.1%	0.21	-
Lipid-modifying agent	62.9%	56.6%	68.6%	69.9%	0.19	0.5%
Daily prescription drugs	8 (5–10)	6 (4–8)	9 (7–11)	12 (9–14)	1.09	-
Bad compliance	62.4%	59.3%	64.6%	70.5%	0.16	0.9%

ACB, Anticholinergic Cognitive Burden score; BMI, body mass index; CKD-REIN, Chronic Kidney Disease – Renal Epidemiology and Information Network Chronic Kidney Disease; eGFR, estimated glomerular filtration rate; ADPKD, autosomal dominant polycystic kidney disease; SMD, standardized mean difference.

The data are reported as the median (interquartile range), the mean ± SD, or a percentage.

A between-group difference was deemed to exist when SMD was > 0.1.

**Table 2:** Medications most frequently prescribed among patients with a high anticholinergic burden (*n* =163)

Anatomical therapeutic and chemical classification		International name	ACB score	*n* (%)
C03	Diuretics	Furosemide	1	95 (58.3%)
N05	Psycholeptics	Hydroxyzine	3	60 (36.8%)
N06	Psychoanaleptics	Paroxetine	2	26 (16.0%)
C07	Beta blocking agents	Atenolol	1	25 (15.3%)
H02	Corticosteroids for systematic use	Prednisone	1	21 (12.9%)
B01	Antithrombotic agents	Warfarin	1	20 (12.3%)
N05	Psycholeptics	Alprazolam	1	15 (9.2%)
N06	Psychoanaleptics	Amitriptyline	3	12 (7.4%)
N02	Analgesics	Codeine	1	12 (7.4%)
A07	Antidiarrheals, intestinal antiinflammatory/antiinfective agents	Loperamide	1	10 (6.1%)
N06	Psychoanaleptics	Clomipramine	3	9 (5.5%)
C01	Cardiac therapy	Digoxin	1	9 (5.5%)
A01	Stomatological preparations	Hydrocortisone	1	6 (3.7%)
C07	Beta blocking agents	Metoprolol	1	6 (3.7%)

ACB score, anticholinergic cognitive burden score.

### Anticholinergic Burden and CKD Progression

During the median (interquartile range) follow-up period of 5.0 (4.6–5.2) years, 647 patients initiated KRT (IR [95% CI]: 5.5 [5.1–6.0]/100 person-yrs); 332 of these had a low or moderate anticholinergic burden (IR [95% CI]: 6.5 [5.8–7.2]/100 person-yrs), and 49 had a high anticholinergic burden (IR [95% CI]: 8.7 [6.3–11.1]/100 person-yrs) (Table 3).

**Table 3:** Incidence rate for CKD progression and the anticholinergic burden (calculated using the ACB score)

Measure	All *N* = 3009	Anticholinergic burden^a^		
		Null *n* = 1453	Low or moderate *n* = 1393	High *n* = 163
Person-yrs	11689.0	6019.9	5106.3	562.8
Number of KRTs	647	266	332	49
Incidence rate for 100 person-yrs	5.5 (5.1–6.0)	4.4 (3.9–4.9)	6.5 (5.8–7.2)	8.7 (6.3–11.1)

ACB score, Anticholinergic Cognitive Burden score; KRT, kidney replacement therapy.

a Anticholinergic burden was calculated using the ACB score.

**Figure 2:**
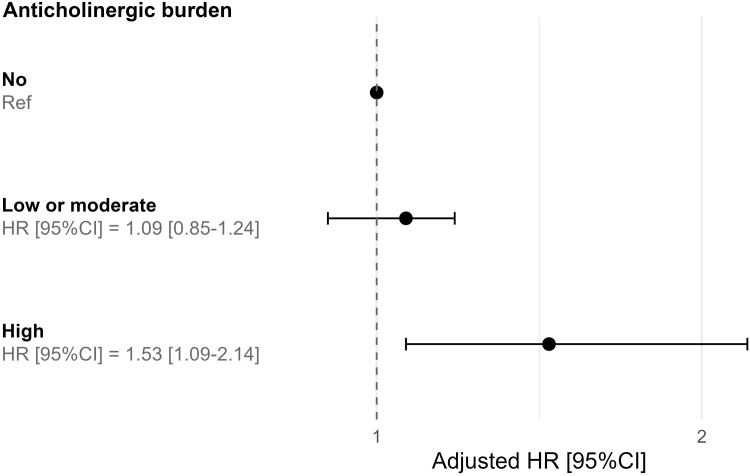
Adjusted hazard ratios for chronic kidney disease progression to kidney replacement therapy and the anticholinergic burden (calculated using the anticholinergic cognitive burden). Hazard ratios were adjusted for age, sex, estimated glomerular filtration, systolic blood pressure (mm Hg), history of cardiovascular disease, diabetes mellitus, dyslipidemia, autosomal dominant polycystic kidney disease, daily prescription drugs, and compliance. CI, confidence interval; HR, hazard ratio.

After adjustment for baseline sociodemographic factors, laboratory variables, comorbidities, and drugs, a high anticholinergic burden was significantly associated with a higher risk of KRT initiation (HR [95% CI]: 1.53 [1.09–2.14]), relative to a null anticholinergic burden. A low or moderate anticholinergic burden was not significantly associated with KRT initiation (HR [95% CI]: 1.09 [0.85–1.24]) (Figure 2).

Similar results were observed when the urinary albumin-to-creatinine ratio was added to the model (Supplementary Table S3).

In a Fine and Gray model accounting for death as a competing event, the HR (95% CI) for KRT initiation was 1.08 (0.91–1.29) for a low or moderate anticholinergic burden and 1.55 (1.11–2.16) for a high anticholinergic burden, relative to a null anticholinergic burden. These estimates were similar to those obtained in the principal analysis (Supplementary Figure S3).

Similar trends (i.e., elevated HRs, particularly for a high anticholinergic burden) were observed with the ARS scale. However, given the smaller subgroup sizes, the CIs were not significant (Supplementary Analysis).

### Anticholinergic Burden and Death

During the median (interquartile range) follow-up period of 5.0 (4.6–5.2) years, 405 patients died (IR [95% CI]: 3.5 [3.1–3.8]/100 person-yrs); 246 of these had a low or moderate anticholinergic burden (IR [95% CI]: 4.8 [4.2–5.4]/100 person-yrs), and 29 had a high anticholinergic burden (IR [95% CI]: 5.2 [3.3–7.0]/100 person-yrs) (Table 4).

**Table 4:** Incidence rate for death and the anticholinergic burden (calculated using the ACB score)

Men				
Measure	All *N* = 1972	Anticholinergic burden^a^		
		No *n* = 956	1-2 *n* = 931	≥ 3 *n* = 85
Person yrs	7625.6	3932.2	3402.1	291.3
Number of deaths	290	104	166	20
Incidence rate for 100 person-yrs	3.8 (3.4–4.2)	2.6 (2.1–3.2)	4.9 (4.1–5.6)	6.9 (3.9–9.9)


Women				
Measure	All *N* = 1037	Anticholinergic burden^a^		
		No *n* = 497	1–2 *n* = 462	≥ 3 *n* = 78
Person yrs	4063.3	2087.7	1704.1	271.5
Number of deaths	115	26	80	9
Incidence rate for 100 person-yrs	2.8 (2.3–3.3)	1.2 (0.8–1.7)	4.7 (3.7–5.7)	3.3 (1.2–5.5)

ACB score, Anticholinergic Cognitive Burden score.

a Anticholinergic burden was calculated using the ACB score.

**Figure 3:**
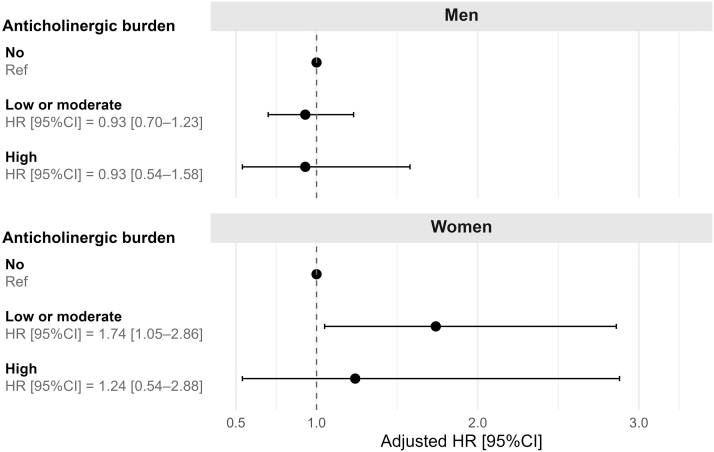
Adjusted hazard ratios for death and the anticholinergic burden (calculated using the anticholinergic cognitive burden). Note: A statistically significant interaction between anticholinergic burden and sex was detected; therefore, analyses were stratified by sex. Hazard ratios were adjusted for age, sex, educational level, smoking status, estimated glomerular filtration rate, urea, uric acid levels, body mass index, history of cardiovascular disease, systolic blood pressure, antihypertensive, lipid-modifying agents, compliance, and the number of prescription drugs taken daily. CI, confidence interval; HR, hazard ratio.

In the analysis of death outcome, a statistically significant interaction between anticholinergic burden and sex was observed (*P* for interaction = 0.03). Accordingly, subgroup analyses were performed. After adjustments for baseline sociodemographic factors, laboratory variables, comorbidities, and drugs, our analysis did not reveal a significant association between the anticholinergic burden and death in men (HR [95% CI]: 0.93 [0.70–1.23] for a low or moderate anticholinergic burden and 0.93 [0.54–1.58] for a high anticholinergic burden), relative to a null anticholinergic burden. Among women, a low or moderate anticholinergic burden was significantly associated with a higher risk of death (HR [95% CI]: 1.74 [1.05–2.86]) but not statistically significant with a high anticholinergic burden (HR [95% CI]: 1.24 [0.54–2.88]), relative to a null anticholinergic burden (Figure 3). Similar results were observed when urinary albumin-to-creatinine ratio was added to the model (Supplementary Table S4). With the ARS scale, the overall estimates (because of the absence of an interaction with sex) were closer to the null and had low statistical power (Supplementary Analysis).

## Discussion

In our cohort of patients with moderate-to-severe CKD being managed by a nephrologist, we found that a high anticholinergic burden was significantly associated with CKD progression (as defined by KRT initiation). The biological plausibility of this association is supported by data from animal models suggesting that the cholinergic system protects kidney function. To the best of our knowledge, the present study is the first to have evaluated the association between the anticholinergic burden and renal outcomes (especially KRT initiation). Several studies have highlighted the role of the cholinergic antiinflammatory pathway (mediated by the vagus nerve and acetylcholine signaling) in reducing kidney inflammation and fibrosis in models of AKI and CKD.^3^^,^^6^ The results of animal experiments have shown that stimulation of the vagus nerve or cholinergic receptors attenuates leukocyte infiltration, cytokine production, and renal tissue damage.^8^^,^^34^ *In vitro* studies have confirmed the presence of muscarinic and nicotinic receptors in key segments of the nephron, including the glomeruli,^10^ inner medullary collecting duct cells,^14^ and proximal tubular epithelial cells.^35^ The *in vitro* studies have also shown that modulation of these receptors (especially the M3 muscarinic subtype) can alter fibrotic and inflammatory responses.^35^ In a context of chronic inflammation, vagal stimulation might protect kidney function and prevent hypertension via the modulation of sympathetic and renin-angiotensin pathways.^13^^,^^34^ These data suggest that cholinergic stimulation in a potential strategy for the management of CKD. However, to the best of our knowledge, the present study is the first to have investigated the opposite phenomenon, namely that medications with anticholinergic properties might have a harmful effect. Our present results suggest that a high anticholinergic burden is associated with CKD progression (defined as initiation of KRT). Although not statistically significant, the association between low or moderate anticholinergic burden indicated an increased risk of KRT (HR > 1), although weaker than that observed with high anticholinergic burden, which may be suggestive of a dose-response relationship between anticholinergic burden and KRT initiation. It should be noted that furosemide contributes 1 point to the score on the ACB scale but does not contribute to the score on the ARS scale. As a result, the prescription of furosemide can shift some patients from a moderate ACB category to a high ACB category. This probably reflects the nature of our cohort, characterized by a high prevalence of cardiovascular comorbidities and the need to manage this risk. Other studies are needed to confirm the presence of a linear association or a threshold effect. Understanding the interplay between the anticholinergic burden and kidney physiology is essential for preserving kidney function and optimizing therapeutic strategies in patients with CKD. Pharmacological management plays a pivotal role in slowing CKD progression, notably through the use of protective agents such as renin-angiotensin system inhibitors and sodium-glucose cotransporter-2 inhibitors.^36^, ^37^, ^38^, ^39^ In this context and with a view to improving CKD outcomes, a reduction in the anticholinergic burden might be a promising therapeutic objective for evaluation in interventional studies. In addition to kidney outcomes, a higher anticholinergic burden has been associated with adverse outcomes in the general population: frailty,^40^ hospital admission, prolongation of hospital stays,^41^ an elevated mortality rate, and a higher risk of major cardiovascular events.^20^ Furthermore, an association between a high anticholinergic burden and cognitive impairment has been consistently demonstrated in both older adults^42^^,^^43^ and patients with CKD.^18^

In the secondary analysis concerning mortality, a significant interaction between sex and anticholinergic burden was found, with no association in men and a positive association between low or moderate burden and mortality in women. Surprisingly, a greater mortality risk was associated with a low or moderate anticholinergic burden but not with a high anticholinergic burden. This might reflect low statistical power for the smaller high anticholinergic burden subgroups; and particularly, the women (*n* = 78, with only 9 events). These sex differences might reflect differences in drug prescription patterns or in underlying pathophysiology mechanisms between men and women. Indeed, in a previous study conducted in the CKD-REIN cohort,^44^ sex-related differences in drug prescription patterns were reported, with women more frequently receiving psychoactive drugs (many of which have a high anticholinergic burden) than men. Differences in the frequency of adverse drug reactions were also noted. Previous epidemiological studies have linked an elevated anticholinergic burden with an increased mortality rate in older adults. For instance, a systematic review found that the majority of high-quality studies reported a positive association between the anticholinergic load and death.^45^ Similarly, a study of nursing home residents with depression found that those exposed to high-level anticholinergic medications had a significantly elevated mortality risk (odds ratio: ∼ 1.3).^46^ However, none of these studies focused specifically on patients with CKD. Prescribing patterns in patients with CKD might differ substantially from those observed in older institutionalized cohorts; for example, exposure to medications with strong anticholinergic properties (particularly antipsychotics and other psychoactive drugs) is less frequent in patients with CKD than in nursing home residents. These drug classes are likely to have a major role in the observed increase in the mortality risk and might account (at least in part) for the difference in the association with mortality between patients with CKD and older, institutionalized adults.

Given the observed association between a high anticholinergic burden and the risk of CKD progression,^14^^,^^16^ and the negative impact, especially on cognitive performance,^15^^,^^18^^,^^21^ deprescribing drugs with strong anticholinergic properties might constitute a strategy for mitigating adverse outcomes. However, the potential benefits of reducing the anticholinergic burden in patients with CKD must be evaluated in other studies.

Our study has several strengths. To the best of our knowledge, the present study is the first to have specifically evaluated the association between the anticholinergic burden and the progression to KRT in patients with CKD. Second, the use of a large, prospective, multicenter cohort of patients under nephrology care provided a robust, nationally representative sample. Third, medication data were collected from physicians’ prescriptions, which ensured high-quality, clinically relevant information. Lastly, we used the validated ACB scale, which is widely used in other studies to quantify the cumulative anticholinergic effect of prescription medications. Our study has several limitations. First, data on over-the-counter medications were not captured, which might have led to underestimation of the true level of anticholinergic drug exposure. Second, although our study sample was large, our subgroup analyses might have lacked the power to detect statistically significant associations in the mortality model, particularly for a high anticholinergic burden. Third, models were adjusted for variables only measured at baseline. However, in a previous study of the CKD-REIN cohort, patient distribution across anticholinergic burden categories did not change markedly over a 5 year of follow-up period.^18^ Fourth, the study’s observational design precluded us from making any causal inferences. Lastly, despite comprehensive direct acyclic graph adjustment, residual confounding by unmeasured factors (e.g., frailty) might have persisted.

In conclusion, our findings suggest that a high anticholinergic burden is associated with an elevated risk of CKD progression in patients with moderate-to-severe CKD, support the hypothesis whereby the disruption of cholinergic signaling could contribute to a decline in kidney function, and highlight the importance of medication reviews and medication optimization in the management of CKD. Further studies are needed to assess whether reducing anticholinergic exposure slows CKD progression.

## Disclosure

NAdP declared financial support from GSK, Boehringer Ingelheim France, Novo Nordisk, Fresenius Medical Care, and Vifor France. All grants were made to the Paris Saclay University. ZAM reported having received grants for CKD-REIN and other research projects from Amgen, Baxter, Fresenius Medical Care, GlaxoSmithKline, Merck Sharp & Dohme-Chibret, Sanofi- Genzyme, Lilly, Otsuka, AstraZeneca, Vifor, and the French government; as well as fees and grants to charities from AstraZeneca, and Boehringer Ingelheim. All the other authors declared no competing interests.

## Appendix

### List of the members of the CKD-REIN Study Group

#### Steering Committee and Coordinators

The CKD-REIN Study Group steering committee and coordinators include Natalia Alencar de Pinho, Dorothée Cannet, Christian Combe, Denis Fouque, Luc Frimat, Aghilès Hamroun, Yves-Edouard Herpe, Christian Jacquelinet, Oriane Lambert, Céline Lange, Maurice Laville, Sophie Liabeuf, Ziad A. Massy, Marie Metzger, Pascal Morel, Christophe Pascal, Roberto Pecoits-Filho, Joost Schantsra, and Bénédicte Stengel.

#### Investigators

Alsace: Professor T. Hannedouche and B. Moulin (CHU, Strasbourg) and Dr A. Klein (CH Colmar); Aquitaine: Professor C. Combe (CHU, Bordeaux), Dr J.P. Bourdenx (Clinique St Augustin, Bordeaux), Dr A. Keller, Dr C. Delclaux (CH, Libourne), Dr B. Vendrely (Clinique St Martin, Pessac), Dr B. Deroure (Clinique Delay, Bayonne), and Dr A. Lacraz (CH, Bayonne); Basse Normandie: Dr T. Lobbedez (CHU, Caen), Dr I. Landru (CH, Lisieux) Ile de France: Professor Z. Massy (CHU, Boulogne – Billancourt), Professor P. Lang (CHU, Créteil), Dr X. Belenfant (CH, Montreuil), Professor E. Thervet (CHU, Paris), Dr P. Urena (Clinique du Landy, St Ouen), and Dr M. Delahousse (Hôpital Foch, Suresnes); Languedoc-Roussillon: Dr C. Vela (CH, Perpignan) Limousin: Professor M. Essig, Dr D. Clément (CHU, Limoges) Lorraine: Dr H. Sekhri, Dr M. Smati (CH, Epinal) Dr M. Jamali, Dr B. Hacq (Clinique Louis Pasteur, Essey-les-Nancy), Dr V. Panescu, Dr M. Bellou (Polyclinique de Gentilly, Nancy), and Professor Luc Frimat (CHU, Vandœuvre-les-Nancy); Midi-Pyrénées: Professor N Kamar (CHU, Toulouse) Nord-Pas-de-Calais: Professors C. Noël and F. Glowacki (CHU, Lille), Dr N. Maisonneuve (CH, Valenciennes), Dr R. Azar (CH, Dunkerque), and Dr M. Hoffmann (Hôpital Professorivé La Louvière, Lille); Pays-de-la Loire: Professor M. Hourmant (CHU, Nantes), Dr A. Testa (Centre de dialyse, Rezé), Dr D. Besnier (CH, St Nazaire) Picardie: Professor G. Choukroun (CHU, Amiens), and Dr G. Lambrey (CH, Beauvais); Professorovence-Alpes - Côte d’Azur: Professor S. Burtey (CHU, Marseille), Dr G. Lebrun (CH, Aix-en-Professorovence), and Dr E. Magnant (Polyclinique du Parc Rambot, Aix-en-Professorovence); Rhône-Alpes: Professor M. Laville, Professor D. Fouque (CHU, Lyon-Sud) and L. Juillard (CHU Edouard Herriot, Lyon), Dr C. Chazot (Centre de rein artificiel Tassin Charcot, Ste Foy-les-Lyon), Professor P. Zaoui (CHU, Grenoble), and Dr F. Kuentz (Centre de santé rénale, Grenoble).

## Supplementary Material

Supplementary File (PDF) **Supplementary Analysis - Results calculated using the ARS. Figure S1.** A directed acyclic graph identifying the minimal sufficient adjustment set for estimating the total effect of the anticholinergic burden on CKD progression (defined as kidney replacement therapy). **Figure S2.** A directed acyclic graph identifying the minimal sufficient adjustment set for estimating the total effect of the anticholinergic burden on death. **Figure S3.** Subdistribution hazard ratios (sHRs) for CKD progression to KRT and the anticholinergic burden, with a Fine and Gray model accounting for death as a competing event. **Table S1.** Definitions of the variables. **Table S2.** Single medications or combinations of medications prescribed to patients with a high anticholinergic burden (*n* = 163). **Table S3.** Adjusted hazard ratios for CKD progression to KRT and the anticholinergic burden (calculated using the ACB) after adding the urinary albumin-to-creatinine ratio (uACR) to the model. **Table S4.** Adjusted hazard ratios for death and the anticholinergic burden (calculated using the ACB) after adding the urinary albumin-to-creatinine ratio (uACR) to the model. STROBE statement.

## Footnotes

Supplementary File (PDF)

**Supplementary Analysis - Results calculated using the ARS.**
**Figure S1.** A directed acyclic graph identifying the minimal sufficient adjustment set for estimating the total effect of the anticholinergic burden on CKD progression (defined as kidney replacement therapy). **Figure S2.** A directed acyclic graph identifying the minimal sufficient adjustment set for estimating the total effect of the anticholinergic burden on death. **Figure S3.** Subdistribution hazard ratios (sHRs) for CKD progression to KRT and the anticholinergic burden, with a Fine and Gray model accounting for death as a competing event. **Table S1.** Definitions of the variables. **Table S2.** Single medications or combinations of medications prescribed to patients with a high anticholinergic burden (*n* = 163). **Table S3.** Adjusted hazard ratios for CKD progression to KRT and the anticholinergic burden (calculated using the ACB) after adding the urinary albumin-to-creatinine ratio (uACR) to the model. **Table S4.** Adjusted hazard ratios for death and the anticholinergic burden (calculated using the ACB) after adding the urinary albumin-to-creatinine ratio (uACR) to the model. **Table S5.** Baseline characteristics of the patients in the CKD-REIN cohort, as a function of the anticholinergic burden (calculated using the ARS). STROBE statement.
