# Supplementary material for: Anticholinergic Burden and Progression to Kidney Replacement Therapy
Source: Kidney Int Rep. 2026 Apr 20;11(7):106552. doi: 10.1016/j.ekir.2026.106552 (PMC13197709; doi:10.1016/j.ekir.2026.106552)
Supplement: Supplementary File (PDF) — Supplementary Analysis - Results calculated using the ARS. Figure S1. A directed acyclic graph identifying the minimal sufficient adjustment set for estimating the total effect of the anticholinergic burden on CKD progression (defined as kidney replacement therapy). Figure S2. A directed acyclic graph identifying the minimal sufficient adjustment set for estimating the total effect of the anticholinergic burden on death. Figure S3. Subdistribution hazard ratios (sHRs) for CKD progression to KRT and the anticholinergic burden, with a Fine and Gray model accounting for death as a competing event. Table S1. Definitions of the variables. Table S2. Single medications or combinations of medications prescribed to patients with a high anticholinergic burden (n = 163). Table S3. Adjusted hazard ratios for CKD progression to KRT and the anticholinergic burden (calculated using the ACB) after adding the urinary albumin-to-creatinine ratio (uACR) to the model. Table S4. Adjusted hazard ratios for death and the anticholinergic burden (calculated using the ACB) after adding the urinary albumin-to-creatinine ratio (uACR) to the model. STROBE statement. [file mmc1.docx]

**SUPPLEMENTARY MATERIAL**

## Supplementary Table 1: Definitions of the variables

| **Variables** | **Definitions** |
| --- | --- |
| Sex | The current registered gender was collected from hospital records as a binary variable (men vs women). Because the hospital records did not distinguish between sex and gender, the terms “man” and “woman” are used consistently throughout the manuscript. |
| Educational level | Educational level was assessed as the number of years in full-time education; in France, a level of 12 years or more corresponds to high school graduation. |
| Compliance | Medication adherence based on the Girerd scale* and was categorized as good compliance (Girerd score = 0) and bas compliance (Girerd score ≥ 3). |
| Dyslipidemia | Dyslipidemia was defined as a history of dyslipidemia or the use of lipid-lowering medication. |
| Diabetes mellitus | Patients were classified as having diabetes mellitus if this condition was reported in their medical records, if they took glucose-lowering medication, or if they had a hemoglobin A1c level ≥6.5%, a fasting glucose level ≥7.0 mmol/L or a random glucose level ≥11.0 mmol/L. |
| Obesity | Obesity was defined as a body mass index ≥30 kg/m². |
| Cardiovascular disease | Cardiovascular disease is defined as a history of:   - Coronary artery disease or - Angina pectoris or - Myocardial infarction or - Coronary artery bypass grafting or - Percutaneous coronary intervention or - Cardiac arrest or - Atrial fibrillation or - Other cardiac arrhythmias or - Implanted pacemaker or - Implanted defibrillator or - Heart failure or - Pulmonary edema or - Pericarditis or - Valvular heart disease or - Cardiac valve prosthesis or - Stroke or - Transient ischemic attack or - Carotid endarterectomy or - Cerebral hemorrhage or - Peripheral vascular disease or - Intermittent claudication or - Arterial bypass surgery/percutaneous intervention for arteritis or - Renal artery stenosis/renal artery surgery or - Aortic aneurysm or - Surgical treatment of an aortic aneurysm. |
| Albumin-to creatinine ratio | The urinary albumin-to-creatinine ratio (ACR) was determined directly or estimated from proteinuria measurements.** We then classified patients according to the Kidney Disease: Improving Global Outcomes 2012 guideline stages***, as follows: A1 (normal): ACR <30mg/g or AER (Albumin Excretion Rate) <30 mg/24h; A2 (high): ACR 30–300mg/g or AER 30–300 mg/24 h; A3 (very high): ACR ≥300 mg/g or AER ≥300 mg/24 h. |

* Girerd X, Hanon O, Anagnostopoulos K, Ciupek C, Mourad JJ, Consoli S. Assessment of antihypertensive compliance using a self-administrated questionnaire: development and use in hypertension clinic. Presse Medicale 2001; 30:1044-8

** Sumida K, Nadkarni GN, Grams ME, et al. Conversion of Urine Protein-Creatinine Ratio or Urine Dipstick Protein to Urine Albumin-Creatinine Ratio for Use in Chronic Kidney Disease Screening and Prognosis : An Individual Participant Based Meta-analysis. Ann Intern Med 2020; 173: 426–35

*** Kidney Disease Improving Global Outcomes CKD Work Group. KDIGO 2012 Clinical Practice Guideline for the Evaluation and Management of Chronic Kidney Disease. Kidney Int Suppl. 2013;(3):1-150

## Supplementary Table 2: Single medications or combinations of medications prescribed to patients with a high anticholinergic burden (n=163)

| **Medication** | **Anticholinergic burden (ACB)** | **Therapeutic classes** | **n (%)** |
| --- | --- | --- | --- |
| hydroxyzine (ACB=3) | 3 | PSYCHOLEPTICS | 14 (8.6%) |
| amitriptyline (ACB=3) | 3 | PSYCHOANALEPTICS | 6 (3.7%) |
| clomipramine (ACB=3) | 3 | PSYCHOANALEPTICS | 5 (3.1%) |
| furosemide (ACB=1) + paroxetine (ACB=2) | 3 | DIURETICS + PSYCHOANALEPTICS | 5 (3.1%) |
| furosemide (ACB=1) + atenolol (ACB=1) + prednisone (ACB=1) | 3 | DIURETICS + BETA BLOCKING AGENTS + CORTICOSTEROIDS FOR SYSTEMIC USE | 4 (2.5%) |
| alprazolam (ACB=1) + paroxetine (ACB=2) | 3 | PSYCHOLEPTICS + PSYCHOANALEPTICS | 3 (1.8%) |
| furosemide (ACB=1) + prednisone (ACB=1) + colchicine (ACB=1) | 3 | DIURETICS + CORTICOSTEROIDS FOR SYSTEMIC USE + ANTIGOUT PREPARATIONS | 3 (1.8%) |
| codeine (ACB=1) + paroxetine (ACB=2) | 3 | ANALGESICS + PSYCHOANALEPTICS | 2 (1.2%) |
| digoxin (ACB=1) + furosemide (ACB=1) + atenolol (ACB=1) | 3 | CARDIAC THERAPY + DIURETICS + BETA BLOCKING AGENTS | 2 (1.2%) |
| loperamide (ACB=1) + furosemide (ACB=1) + alprazolam (ACB=1) | 3 | ANTIDIARRHEALS, INTESTINAL ANTIINFLAMMATORY/ANTIINFECTIVE AGENTS + DIURETICS + PSYCHOLEPTICS | 2 (1.2%) |
| ranitidine (ACB=1) + furosemide (ACB=1) + atenolol (ACB=1) | 3 | DRUGS FOR ACID RELATED DISORDERS + DIURETICS + BETA BLOCKING AGENTS | 2 (1.2%) |
| warfarin (ACB=1) + furosemide (ACB=1) + atenolol (ACB=1) | 3 | ANTITHROMBOTIC AGENTS + DIURETICS + BETA BLOCKING AGENTS | 2 (1.2%) |
| warfarin (ACB=1) + furosemide (ACB=1) + codeine (ACB=1) | 3 | ANTITHROMBOTIC AGENTS + DIURETICS + ANALGESICS | 2 (1.2%) |
| warfarin (ACB=1) + furosemide (ACB=1) + prednisone (ACB=1) | 3 | ANTITHROMBOTIC AGENTS + DIURETICS + CORTICOSTEROIDS FOR SYSTEMIC USE | 2 (1.2%) |
| alverine (ACB=1) + furosemide (ACB=1) + atenolol and nifedipine (ACB=1) | 3 | DRUGS FOR FUNCTIONAL GASTROINTESTINAL DISORDERS + DIURETICS + BETA BLOCKING AGENTS | 1 (0.6%) |
| alverine (ACB=1) + furosemide (ACB=1) + colchicine (ACB=1) | 3 | DRUGS FOR FUNCTIONAL GASTROINTESTINAL DISORDERS + DIURETICS + ANTIGOUT PREPARATIONS | 1 (0.6%) |
| alverine (ACB=1) + furosemide (ACB=1) + hydrocortisone (ACB=1) | 3 | DRUGS FOR FUNCTIONAL GASTROINTESTINAL DISORDERS + DIURETICS + CORTICOSTEROIDS FOR SYSTEMIC USE | 1 (0.6%) |
| atenolol (ACB=1) + carbamazepine (ACB=2) | 3 | BETA BLOCKING AGENTS + ANTIEPILEPTICS | 1 (0.6%) |
| digoxin (ACB=1) + furosemide (ACB=1) + hydrocortisone (ACB=1) | 3 | CARDIAC THERAPY + DIURETICS + CORTICOSTEROIDS FOR SYSTEMIC USE | 1 (0.6%) |
| digoxin (ACB=1) + furosemide (ACB=1) + theophylline (ACB=1) | 3 | CARDIAC THERAPY + DIURETICS + DRUGS FOR OBSTRUCTIVE AIRWAY DISEASES | 1 (0.6%) |
| dihydrocodeine (ACB=1) + paroxetine (ACB=2) | 3 | ANALGESICS + PSYCHOANALEPTICS | 1 (0.6%) |
| furosemide (ACB=1) + atenolol (ACB=1) + alprazolam (ACB=1) | 3 | DIURETICS + BETA BLOCKING AGENTS + PSYCHOLEPTICS | 1 (0.6%) |
| furosemide (ACB=1) + atenolol (ACB=1) + codeine (ACB=1) | 3 | DIURETICS + BETA BLOCKING AGENTS + ANALGESICS | 1 (0.6%) |
| furosemide (ACB=1) + atenolol (ACB=1) + hydrocortisone (ACB=1) | 3 | DIURETICS + BETA BLOCKING AGENTS + CORTICOSTEROIDS FOR SYSTEMIC USE | 1 (0.6%) |
| furosemide (ACB=1) + hydrochlorothiazide and potassium-sparing agents (ACB=1) + atenolol (ACB=1) | 3 | DIURETICS + DIURETICS + BETA BLOCKING AGENTS | 1 (0.6%) |
| furosemide (ACB=1) + metoprolol (ACB=1) + codeine (ACB=1) | 3 | DIURETICS + BETA BLOCKING AGENTS + COUGH AND COLD PREPARATIONS | 1 (0.6%) |
| furosemide (ACB=1) + metoprolol and felodipine (ACB=1) + prednisone (ACB=1) | 3 | DIURETICS + BETA BLOCKING AGENTS + CORTICOSTEROIDS FOR SYSTEMIC USE | 1 (0.6%) |
| furosemide (ACB=1) + prednisone (ACB=1) + morphine (ACB=1) | 3 | DIURETICS + CORTICOSTEROIDS FOR SYSTEMIC USE + ANALGESICS | 1 (0.6%) |
| hydrocortisone (ACB=1) + carbamazepine (ACB=2) | 3 | CORTICOSTEROIDS FOR SYSTEMIC USE + ANTIEPILEPTICS | 1 (0.6%) |
| isosorbide (ACB=1) + furosemide (ACB=1) + theophylline (ACB=1) | 3 | CARDIAC THERAPY + DIURETICS + DRUGS FOR OBSTRUCTIVE AIRWAY DISEASES | 1 (0.6%) |
| isosorbide (ACB=1) + paroxetine (ACB=2) | 3 | CARDIAC THERAPY + PSYCHOANALEPTICS | 1 (0.6%) |
| loperamide (ACB=1) + furosemide (ACB=1) + atenolol (ACB=1) | 3 | ANTIDIARRHEALS, INTESTINAL ANTIINFLAMMATORY/ANTIINFECTIVE AGENTS + DIURETICS + BETA BLOCKING AGENTS | 1 (0.6%) |
| loperamide (ACB=1) + furosemide (ACB=1) + colchicine (ACB=1) | 3 | ANTIDIARRHEALS, INTESTINAL ANTIINFLAMMATORY/ANTIINFECTIVE AGENTS + DIURETICS + ANTIGOUT PREPARATIONS | 1 (0.6%) |
| loperamide (ACB=1) + paroxetine (ACB=2) | 3 | ANTIDIARRHEALS, INTESTINAL ANTIINFLAMMATORY/ANTIINFECTIVE AGENTS + PSYCHOANALEPTICS | 1 (0.6%) |
| metoprolol (ACB=1) + colchicine (ACB=1) + alprazolam (ACB=1) | 3 | BETA BLOCKING AGENTS + ANTIGOUT PREPARATIONS + PSYCHOLEPTICS | 1 (0.6%) |
| olanzapine (ACB=3) | 3 | PSYCHOLEPTICS | 1 (0.6%) |
| paroxetine (ACB=2) + alimemazine (ACB=1) | 3 | PSYCHOANALEPTICS + ANTIHISTAMINES FOR SYSTEMIC USE | 1 (0.6%) |
| prednisone (ACB=1) + hydrocortisone (ACB=1) + alprazolam (ACB=1) | 3 | CORTICOSTEROIDS FOR SYSTEMIC USE + CORTICOSTEROIDS FOR SYSTEMIC USE + PSYCHOLEPTICS | 1 (0.6%) |
| quetiapine (ACB=3) | 3 | PSYCHOLEPTICS | 1 (0.6%) |
| ranitidine (ACB=1) + loperamide (ACB=1) + furosemide (ACB=1) | 3 | DRUGS FOR ACID RELATED DISORDERS + ANTIDIARRHEALS, INTESTINAL ANTIINFLAMMATORY/ANTIINFECTIVE AGENTS + DIURETICS | 1 (0.6%) |
| trihexyphenidyl (ACB=3) | 3 | ANTI-PARKINSON DRUGS | 1 (0.6%) |
| trimipramine (ACB=3) | 3 | PSYCHOANALEPTICS | 1 (0.6%) |
| warfarin (ACB=1) + digoxin (ACB=1) + furosemide (ACB=1) | 3 | ANTITHROMBOTIC AGENTS + CARDIAC THERAPY + DIURETICS | 1 (0.6%) |
| warfarin (ACB=1) + furosemide (ACB=1) + clorazepate (ACB=1) | 3 | ANTITHROMBOTIC AGENTS + DIURETICS + PSYCHOLEPTICS | 1 (0.6%) |
| furosemide (ACB=1) + hydroxyzine (ACB=3) | 4 | DIURETICS + PSYCHOLEPTICS | 20 (12.3%) |
| colchicine (ACB=1) + hydroxyzine (ACB=3) | 4 | ANTIGOUT PREPARATIONS + PSYCHOLEPTICS | 3 (1.8%) |
| furosemide (ACB=1) + amitriptyline (ACB=3) | 4 | DIURETICS + PSYCHOANALEPTICS | 2 (1.2%) |
| furosemide (ACB=1) + atenolol (ACB=1) + paroxetine (ACB=2) | 4 | DIURETICS + BETA BLOCKING AGENTS + PSYCHOANALEPTICS | 2 (1.2%) |
| warfarin (ACB=1) + furosemide (ACB=1) + paroxetine (ACB=2) | 4 | ANTITHROMBOTIC AGENTS + DIURETICS + PSYCHOANALEPTICS | 2 (1.2%) |
| alprazolam (ACB=1) + hydroxyzine (ACB=3) | 4 | PSYCHOLEPTICS + PSYCHOLEPTICS | 1 (0.6%) |
| alverine (ACB=1) + furosemide (ACB=1) + colchicine (ACB=1) + codeine (ACB=1) | 4 | DRUGS FOR FUNCTIONAL GASTROINTESTINAL DISORDERS + DIURETICS + ANTIGOUT PREPARATIONS + ANALGESICS | 1 (0.6%) |
| apomorphine (ACB=1) + clozapine (ACB=3) | 4 | ANTI-PARKINSON DRUGS + PSYCHOLEPTICS | 1 (0.6%) |
| atenolol and nifedipine (ACB=1) + alprazolam (ACB=1) + paroxetine (ACB=2) | 4 | BETA BLOCKING AGENTS + PSYCHOLEPTICS + PSYCHOANALEPTICS | 1 (0.6%) |
| clorazepate (ACB=1) + clomipramine (ACB=3) | 4 | PSYCHOLEPTICS + PSYCHOANALEPTICS | 1 (0.6%) |
| codeine (ACB=1) + hydroxyzine (ACB=3) | 4 | ANALGESICS + PSYCHOLEPTICS | 1 (0.6%) |
| colchicine (ACB=1) + amitriptyline (ACB=3) | 4 | ANTIGOUT PREPARATIONS + PSYCHOANALEPTICS | 1 (0.6%) |
| colchicine (ACB=1) + clomipramine (ACB=3) | 4 | ANTIGOUT PREPARATIONS + PSYCHOANALEPTICS | 1 (0.6%) |
| furosemide (ACB=1) + alprazolam (ACB=1) + paroxetine (ACB=2) | 4 | DIURETICS + PSYCHOLEPTICS + PSYCHOANALEPTICS | 1 (0.6%) |
| furosemide (ACB=1) + atenolol (ACB=1) + prednisone (ACB=1) + alprazolam (ACB=1) | 4 | DIURETICS + BETA BLOCKING AGENTS + CORTICOSTEROIDS FOR SYSTEMIC USE + PSYCHOLEPTICS | 1 (0.6%) |
| furosemide (ACB=1) + metoprolol (ACB=1) + paroxetine (ACB=2) | 4 | DIURETICS + BETA BLOCKING AGENTS + PSYCHOANALEPTICS | 1 (0.6%) |
| furosemide (ACB=1) + oxybutynin (ACB=3) | 4 | DIURETICS + UROLOGICALS | 1 (0.6%) |
| loperamide (ACB=1) + digoxin (ACB=1) + furosemide (ACB=1) + colchicine (ACB=1) | 4 | ANTIDIARRHEALS, INTESTINAL ANTIINFLAMMATORY/ANTIINFECTIVE AGENTS + CARDIAC THERAPY + DIURETICS + ANTIGOUT PREPARATIONS | 1 (0.6%) |
| loperamide (ACB=1) + hydroxyzine (ACB=3) | 4 | ANTIDIARRHEALS, INTESTINAL ANTIINFLAMMATORY/ANTIINFECTIVE AGENTS + PSYCHOLEPTICS | 1 (0.6%) |
| metoprolol (ACB=1) + captopril (ACB=1) + paroxetine (ACB=2) | 4 | BETA BLOCKING AGENTS + AGENTS ACTING ON THE RENIN-ANGIOTENSIN SYSTEM + PSYCHOANALEPTICS | 1 (0.6%) |
| warfarin (ACB=1) + digoxin (ACB=1) + furosemide (ACB=1) + prednisone (ACB=1) | 4 | ANTITHROMBOTIC AGENTS + CARDIAC THERAPY + DIURETICS + CORTICOSTEROIDS FOR SYSTEMIC USE | 1 (0.6%) |
| warfarin (ACB=1) + furosemide (ACB=1) + atenolol (ACB=1) + alprazolam (ACB=1) | 4 | ANTITHROMBOTIC AGENTS + DIURETICS + BETA BLOCKING AGENTS + PSYCHOLEPTICS | 1 (0.6%) |
| warfarin (ACB=1) + furosemide (ACB=1) + carbamazepine (ACB=2) | 4 | ANTITHROMBOTIC AGENTS + DIURETICS + ANTIEPILEPTICS | 1 (0.6%) |
| warfarin (ACB=1) + hydroxyzine (ACB=3) | 4 | ANTITHROMBOTIC AGENTS + PSYCHOLEPTICS | 1 (0.6%) |
| warfarin (ACB=1) + prednisone (ACB=1) + paroxetine (ACB=2) | 4 | ANTITHROMBOTIC AGENTS + CORTICOSTEROIDS FOR SYSTEMIC USE + PSYCHOANALEPTICS | 1 (0.6%) |
| furosemide (ACB=1) + prednisone (ACB=1) + hydroxyzine (ACB=3) | 5 | DIURETICS + CORTICOSTEROIDS FOR SYSTEMIC USE + PSYCHOLEPTICS | 2 (1.2%) |
| digoxin (ACB=1) + furosemide (ACB=1) + atenolol (ACB=1) + carbamazepine (ACB=2) | 5 | CARDIAC THERAPY + DIURETICS + BETA BLOCKING AGENTS + ANTIEPILEPTICS | 1 (0.6%) |
| digoxin (ACB=1) + furosemide (ACB=1) + hydroxyzine (ACB=3) | 5 | CARDIAC THERAPY + DIURETICS + PSYCHOLEPTICS | 1 (0.6%) |
| disopyramide (ACB=1) + furosemide (ACB=1) + levomepromazine (ACB=2) + clorazepate (ACB=1) | 5 | CARDIAC THERAPY + DIURETICS + PSYCHOLEPTICS + PSYCHOLEPTICS | 1 (0.6%) |
| furosemide (ACB=1) + atenolol (ACB=1) + hydroxyzine (ACB=3) | 5 | DIURETICS + BETA BLOCKING AGENTS + PSYCHOLEPTICS | 1 (0.6%) |
| furosemide (ACB=1) + atenolol (ACB=1) + oxybutynin (ACB=3) | 5 | DIURETICS + BETA BLOCKING AGENTS + UROLOGICALS | 1 (0.6%) |
| furosemide (ACB=1) + codeine (ACB=1) + hydroxyzine (ACB=3) | 5 | DIURETICS + ANALGESICS + PSYCHOLEPTICS | 1 (0.6%) |
| furosemide (ACB=1) + prednisone (ACB=1) + amitriptyline (ACB=3) | 5 | DIURETICS + CORTICOSTEROIDS FOR SYSTEMIC USE + PSYCHOANALEPTICS | 1 (0.6%) |
| hydrocortisone (ACB=1) + colchicine (ACB=1) + hydroxyzine (ACB=3) | 5 | CORTICOSTEROIDS FOR SYSTEMIC USE + ANTIGOUT PREPARATIONS + PSYCHOLEPTICS | 1 (0.6%) |
| hydroxyzine (ACB=3) + paroxetine (ACB=2) | 5 | PSYCHOLEPTICS + PSYCHOANALEPTICS | 1 (0.6%) |
| metoprolol (ACB=1) + captopril (ACB=1) + meclozine (ACB=3) | 5 | BETA BLOCKING AGENTS + AGENTS ACTING ON THE RENIN-ANGIOTENSIN SYSTEM + ANTIHISTAMINES FOR SYSTEMIC USE | 1 (0.6%) |
| ranitidine (ACB=1) + oxybutynin (ACB=3) + alprazolam (ACB=1) | 5 | DRUGS FOR ACID RELATED DISORDERS + UROLOGICALS + PSYCHOLEPTICS | 1 (0.6%) |
| warfarin (ACB=1) + furosemide (ACB=1) + amitriptyline (ACB=3) | 5 | ANTITHROMBOTIC AGENTS + DIURETICS + PSYCHOANALEPTICS | 1 (0.6%) |
| warfarin (ACB=1) + furosemide (ACB=1) + hydroxyzine (ACB=3) | 5 | ANTITHROMBOTIC AGENTS + DIURETICS + PSYCHOLEPTICS | 1 (0.6%) |
| warfarin (ACB=1) + furosemide (ACB=1) + prednisone (ACB=1) + hydroxyzine (ACB=3) | 6 | ANTITHROMBOTIC AGENTS + DIURETICS + CORTICOSTEROIDS FOR SYSTEMIC USE + PSYCHOLEPTICS | 2 (1.2%) |
| chlorpromazine (ACB=3) + quetiapine (ACB=3) | 6 | PSYCHOLEPTICS + PSYCHOLEPTICS | 1 (0.6%) |
| fentanyl (ACB=1) + hydroxyzine (ACB=3) + paroxetine (ACB=2) | 6 | ANALGESICS + PSYCHOLEPTICS + PSYCHOANALEPTICS | 1 (0.6%) |
| furosemide (ACB=1) + atenolol (ACB=1) + prednisone (ACB=1) + hydroxyzine (ACB=3) | 6 | DIURETICS + BETA BLOCKING AGENTS + CORTICOSTEROIDS FOR SYSTEMIC USE + PSYCHOLEPTICS | 1 (0.6%) |
| furosemide (ACB=1) + atenolol (ACB=1) + risperidone (ACB=1) + hydroxyzine (ACB=3) | 6 | DIURETICS + BETA BLOCKING AGENTS + PSYCHOLEPTICS + PSYCHOLEPTICS | 1 (0.6%) |
| furosemide (ACB=1) + colchicine (ACB=1) + hydroxyzine (ACB=3) + theophylline (ACB=1) | 6 | DIURETICS + ANTIGOUT PREPARATIONS + PSYCHOLEPTICS + DRUGS FOR OBSTRUCTIVE AIRWAY DISEASES | 1 (0.6%) |
| loperamide (ACB=1) + furosemide (ACB=1) + colchicine (ACB=1) + hydroxyzine (ACB=3) | 6 | ANTIDIARRHEALS, INTESTINAL ANTIINFLAMMATORY/ANTIINFECTIVE AGENTS + DIURETICS + ANTIGOUT PREPARATIONS + PSYCHOLEPTICS | 1 (0.6%) |
| loperamide (ACB=1) + warfarin (ACB=1) + furosemide (ACB=1) + hydroxyzine (ACB=3) | 6 | ANTIDIARRHEALS, INTESTINAL ANTIINFLAMMATORY/ANTIINFECTIVE AGENTS + ANTITHROMBOTIC AGENTS + DIURETICS + PSYCHOLEPTICS | 1 (0.6%) |
| olanzapine (ACB=3) + hydroxyzine (ACB=3) | 6 | PSYCHOLEPTICS + PSYCHOLEPTICS | 1 (0.6%) |
| quetiapine (ACB=3) + trimipramine (ACB=3) | 6 | PSYCHOLEPTICS + PSYCHOANALEPTICS | 1 (0.6%) |
| codeine (ACB=1) + oxcarbazepine (ACB=2) + alprazolam (ACB=1) + hydroxyzine (ACB=3) | 7 | ANALGESICS + ANTIEPILEPTICS + PSYCHOLEPTICS + PSYCHOLEPTICS | 1 (0.6%) |
| morphine (ACB=1) + hydroxyzine (ACB=3) + amitriptyline (ACB=3) | 7 | ANALGESICS + PSYCHOLEPTICS + PSYCHOANALEPTICS | 1 (0.6%) |
| codeine (ACB=1) + clorazepate (ACB=1) + imipramine (ACB=3) + clomipramine (ACB=3) | 8 | ANALGESICS + PSYCHOLEPTICS + PSYCHOANALEPTICS + PSYCHOANALEPTICS | 1 (0.6%) |
| olanzapine (ACB=3) + clomipramine (ACB=3) + paroxetine (ACB=2) | 8 | PSYCHOLEPTICS + PSYCHOANALEPTICS + PSYCHOANALEPTICS | 1 (0.6%) |

## Supplementary Table 3: Adjusted hazard ratios for CKD progression to KRT and the anticholinergic burden (calculated using the ACB) after adding the urinary albumin-to-creatinine ratio (uACR) to the model

|  | **Model 1: Principal model** | **Model 1 + uACR** |
| --- | --- | --- |
| Null ACB | *Ref* | *Ref* |
| Low or moderate ACB | 1.09 [0.85-1.24] | 1.01 [0.83 – 1.21] |
| High ACB | 1.53 [1.09-2.14] | 1.48 [1.06 – 2.08] |

ACB, Anticholinergic cognitive burden; uACR, urinary albumin-to-creatinine ratio.

## Supplementary Table 4: Adjusted hazard ratios for death and the anticholinergic burden (calculated using the ACB) after adding the urinary albumin-to-creatinine ratio (uACR) to the model

|  | **Men** | | **Women** | |
| --- | --- | --- | --- | --- |
|  | **Principal analysis** | **With uACR** | **Principal analysis** | **With uACR** |
| Null ACB | *Ref* | *Ref* | *Ref* | *Ref* |
| Low or moderate ACB | 0.93 [0.70-1.23] | 0.90 [0.68-1.20] | 1.74 [1.05-2.86] | 1.75 [1.06-2.88] |
| High ACB | 0.93 [0.54-1.58] | 0.90 [0.53-1.53] | 1.24 [0.54-2.88] | 1.25 [0.54-2.93] |

ACB, Anticholinergic cognitive burden; uACR, urinary albumin-to-creatinine ratio.

## Supplementary Figure 1: A directed acyclic graph identifying the minimal sufficient adjustment set for estimating the total effect of the anticholinergic burden on CKD progression (defined as kidney replacement therapy).


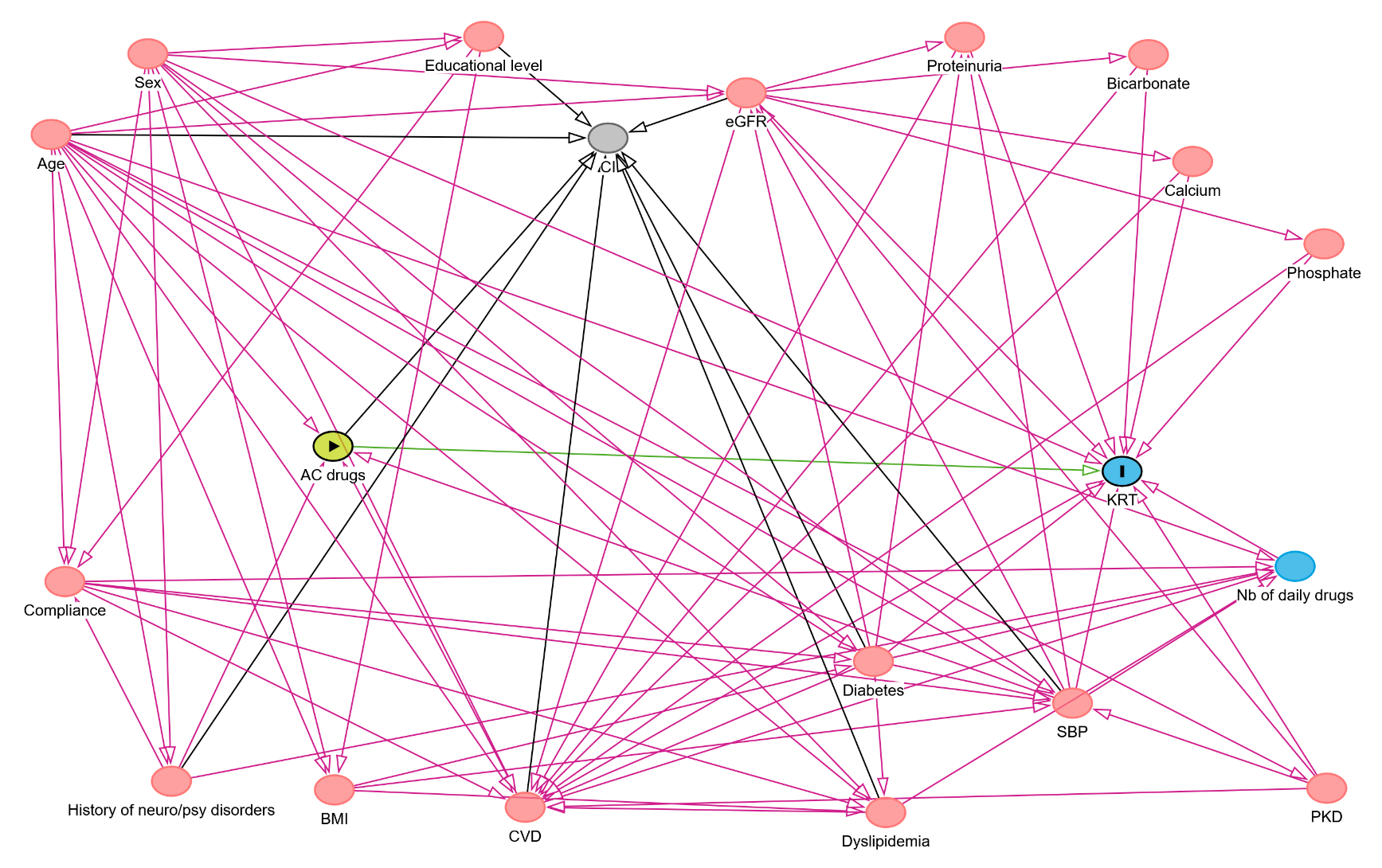


AC drugs, anticholinergic drugs; KRT, kidney replacement therapy; CI, cognitive impairment; eGFR, estimate glomerular filtration rate; Nb, number; neuro/psy disorders, neurological and/or psychiatric disorders; BMI, body mass index; CVD, cardiovascular disease; SBP, systolic blood pressure; PKD, autosomal dominant polycystic kidney disease.

Note: The minimal sufficient adjustment set for estimating the total effect of anticholinergic drugs on kidney replacement therapy was: age, sex, history of cardiovascular disease, diabetes mellitus, dyslipidemia, systolic blood pressure, autosomal dominant polycystic kidney disease, number of daily drugs and compliance. Given the nature of our study, estimated glomerular filtration rate (mL/min/1.73m²) was additionally included.

## Supplementary Figure 2: A directed acyclic graph identifying the minimal sufficient adjustment set for estimating the total effect of the anticholinergic burden on death.


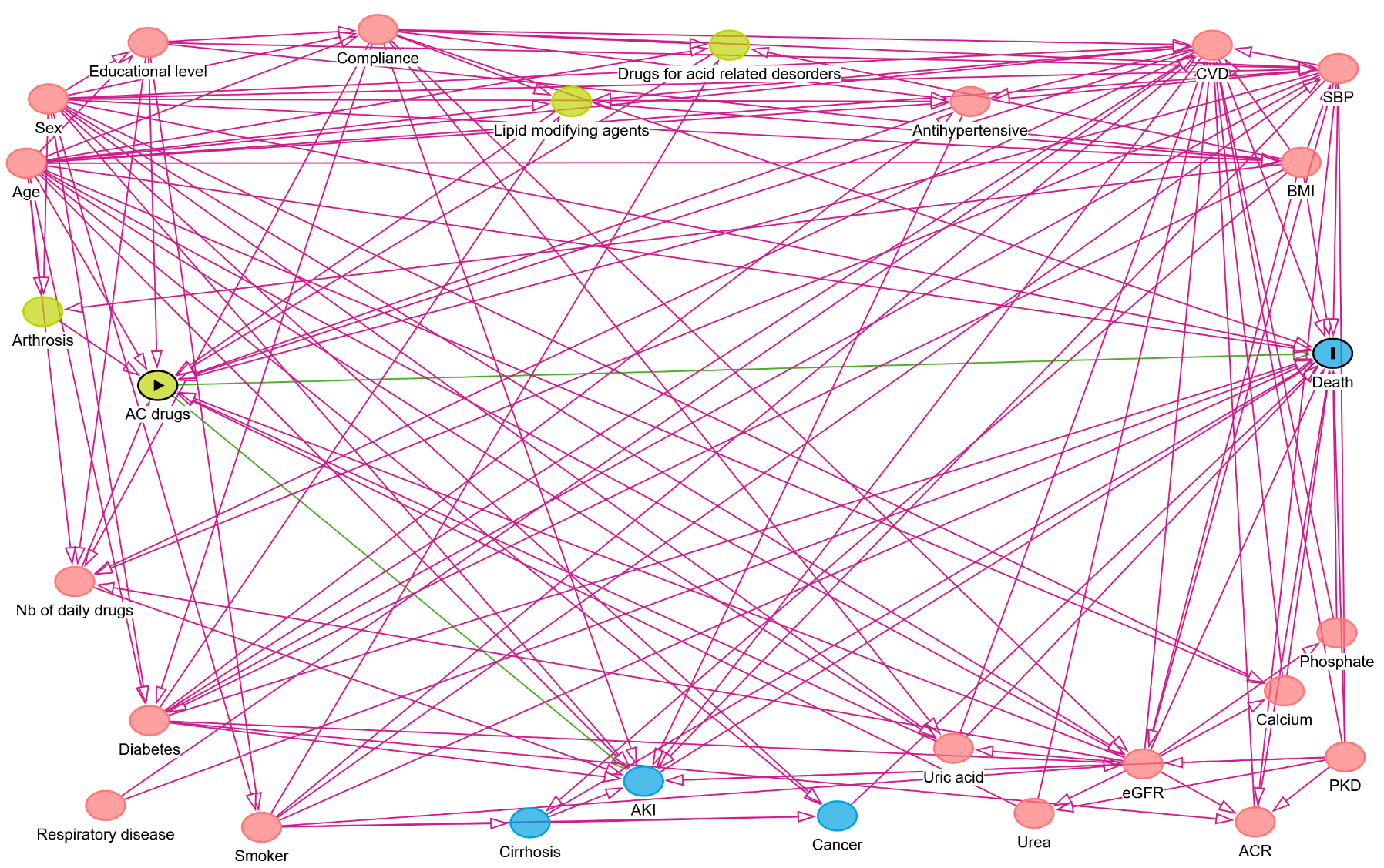


AC drugs, anticholinergic drugs; eGFR, estimate glomerular filtration rate; Nb, number; BMI, body mass index; CVD, cardiovascular disease; SBP, systolic blood pressure; AKI, acute kidney injury; PKD, autosomal dominant polycystic kidney disease

Note: The minimal sufficient adjustment set for estimating the total effect of anticholinergic drugs on death was: age, sex, educational level, smoking status, estimated glomerular filtration rate, urea, uric acid, body mass index, systolic blood pressure, history of cardiovascular disease, use of antihypertensive drugs and lipid modifying agents, number of daily drugs and compliance.

## Supplementary Figure 3: Subdistribution hazard ratios (sHRs) for CKD progression to KRT and the anticholinergic burden, with a Fine and Gray model accounting for death as a competing event


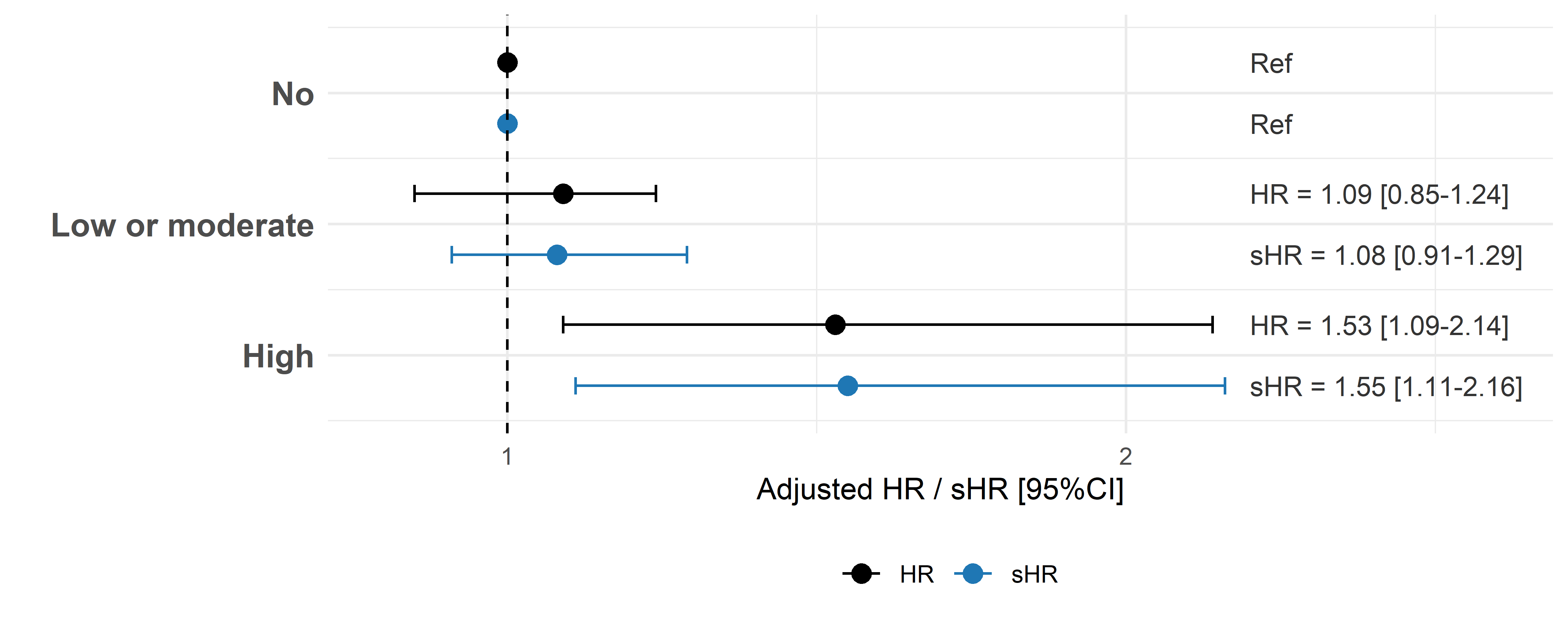


HR, hazard ratio; CI, confidence interval; sHR, subdistribution hazard ratios.

Hazard ratios were adjusted for age, sex, eGFR, systolic blood pressure (mmHg), history of cardiovascular disease, diabetes mellitus, dyslipidemia, autosomal dominant polycystic kidney disease, the number of prescription drugs taken daily, and compliance.

sHR accounting for death as a competing event.

# SUPPLEMENTARY ANALYSIS: Results calculated using Anticholinergic Risk Scale (ARS)

*Baseline characteristics*

Of the 3009 of the CKD-REIN cohort, only 90 (3%) patients had a high anticholinergic burden according to the ARS (vs. 163 (5%) using the ACB scale. Hence, the ARS captured fewer drugs with anticholinergic properties than the ACB scale did (**Table SM1**).

The majority (67%) of the patients with a high anticholinergic burden were receiving a psycholeptic with an ARS score of three, 13% were receiving a psychoanaleptic with an ARS score of three, and 12% were receiving an antihistamine with an ARS score of two (**Table SM2**). When we focused on drug combinations, 51% of the patients with a high anticholinergic burden were receiving hydroxyzine (ARS=3), 11% were receiving amitryptyline (ARS=3) or hydroxyzine (ARS=3), and 3% were receiving loperamide (ARS=2) (**Figure SM1**). Using the ARS score, patients with a high anticholinergic burden were more likely to be receiving medications with a high score or a combination of a medication with a moderate score and a medication with a high score, compared with the results for the ACB scale.

## Table SM1: Baseline characteristics of the patients in the CKD-REIN cohort, as a function of the anticholinergic burden (calculated using the ARS)

|  | **All** (n=3,009) | **Anticholinergic burden** | | | **SMD** | **Imputed data** (%) |
| --- | --- | --- | --- | --- | --- | --- |
|  |  | **Null** (n=2,745) | **Low or moderate** (n=174) | **High** (n=90) |  |  |
| **Sociodemographic factors** |  |  |  |  |  |  |
| Age (years) | 69 [60-76] | 69 [60-77] | 68 [62-75] | 71.5 [64-77] | 0.12 | - |
| Men | 65.5% | 66.5% | 56.9% | 52.2% | 0.20 | - |
| Educational level |  |  |  |  | 0.43 | 1.2% |
| < 9 years | 14.8% | 14.5% | 19.1% | 15.8% |  |  |
| 9-11 years | 49.2% | 48.5% | 48.1% | 71.6% |  |  |
| ≥ 12 years | 36.0% | 37.0% | 32.9% | 12.6% |  |  |
| Smoking status |  |  |  |  | 0.15 | 0.8% |
| Current smoker | 11.8% | 12.0% | 8.1% | 12.2% |  |  |
| Ex-smoker | 47.0% | 47.1% | 48.0% | 40.3% |  |  |
| Non-smoker | 41.2% | 40.8% | 44.0% | 47.5% |  |  |
| **Clinical and laboratory variables** |  |  |  |  |  |  |
| Obesity (BMI ≥30 kg/m²) | 35.5% | 34.7% | 43.2% | 45.9% | 0.17 | 2.1% |
| Systolic blood pressure  (mmHg) | 141.5 ± 19.7 | 141.6 ± 19.7 | 139.1 ± 19 | 143.1 ± 20.2 | 0.14 | 0.6% |
| eGFR (mL/min/1.73 m²) | 34.0 ± 13.0 | 34.0 ± 13.0 | 34.2 ± 13.6 | 33.1 ± 10.9 | 0.12 | - |
| Urinary albumin-to-creatinine  ratio (mg/g) |  |  |  |  |  | 11.9% |
| A1 (< 30 mg/g) | 30.2% | 30.2% | 31.1% | 26.9% |  |  |
| A2 (30-300 mg/g) | 35.3% | 35.6% | 33.8% | 28.9% |  |  |
| A3 (≥ 300 mg/g) | 34.5% | 34.2% | 35.1% | 44.2% |  |  |
| Urea (mmol/L) | 12.8 [9.7-17.1] | 12.8 [9.7-17] | 12.6 [9.4-17.6] | 13 [10-19.3] | 0.17 | 4.2% |
| **Medical history** |  |  |  |  |  |  |
| Cardiovascular disease | 53.1% | 52.8% | 55.4% | 59.4% | 0.09 | 1.3% |
| Diabetes mellitus | 43.4% | 42.3% | 52.1% | 61.1% | 0.26 | 0.2% |
| Dyslipidemia | 73.7% | 73.7% | 71.3% | 76.9% | 0.09 | 0.2% |
| PKD | 5.8% | 5.7% | 7.3% | 3.5% | 0.12 | 5.9% |
| **Medication use** |  |  |  |  |  |  |
| Antihypertensive agent | 93.2% | 93.3% | 92.0% | 94.4% | 0.07 | - |
| Lipid-modifying agent | 62.9% | 62.8% | 62.6% | 66.7% | 0.06 | 0.5% |
| Number of prescription drugs taken daily | 8 [5-10] | 7 [5-10] | 9 [7-13] | 11 [9-14] | 0.72 | - |
| Compliance | 62.4% | 62.2% | 64.0% | 65.6% | 0.05 | 0.9% |

SMD, standardized mean difference; BMI, body mass index; eGFR, estimated glomerular filtration rate; PKD, autosomal dominant polycystic kidney disease

Note: The data are reported as the median [interquartile range], the mean ± SD, or a percentage.

A between-group difference was deemed to exist when SMD was > 0.1.


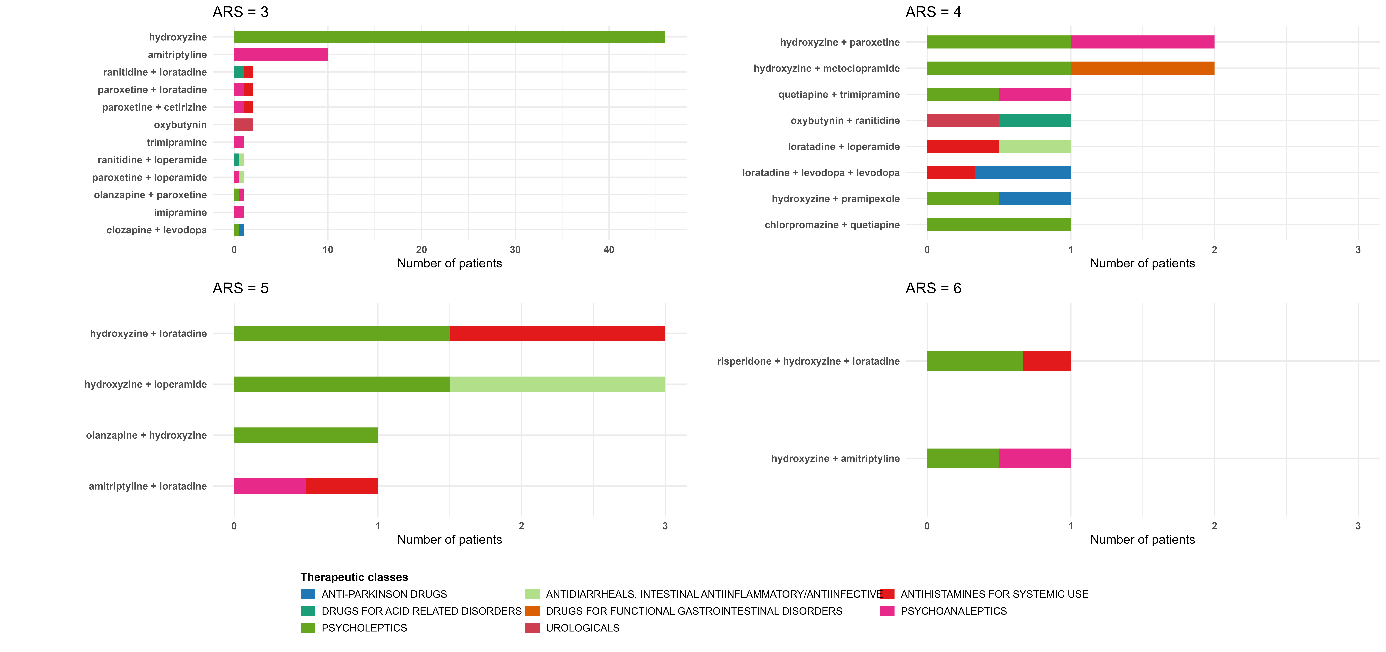


## Figure SM1: Medications or combinations of medications leading to a high anticholinergic burden (ARS score ≥ 3)

## Table SM2: Medications or combinations of medications prescribed among patients with a high anticholinergic burden (n=90)

| **Medication** | **ATC** | **n (%)** |
| --- | --- | --- |
| amitriptyline (ARS=3) | PSYCHOANALEPTICS | 10 (11.1%) |
| hydroxyzine (ARS=3) | PSYCHOLEPTICS | 46 (51.1%) |
| imipramine (ARS=3) | PSYCHOANALEPTICS | 1 (1.1%) |
| levodopa (ARS=1) + clozapine (ARS=2) | ANTI-PARKINSON DRUGS + PSYCHOLEPTICS | 1 (1.1%) |
| loperamide (ARS=2) + paroxetine (ARS=1) | ANTIDIARRHEALS, INTESTINAL ANTIINFLAMMATORY/ANTIINFECTIVE AGENTS + PSYCHOANALEPTICS | 1 (1.1%) |
| olanzapine (ARS=2) + paroxetine (ARS=1) | PSYCHOLEPTICS + PSYCHOANALEPTICS | 1 (1.1%) |
| oxybutynin (ARS=3) | UROLOGICALS | 2 (2.2%) |
| paroxetine (ARS=1) + cetirizine (ARS=2) | PSYCHOANALEPTICS + ANTIHISTAMINES FOR SYSTEMIC USE | 2 (2.2%) |
| paroxetine (ARS=1) + loratadine (ARS=2) | PSYCHOANALEPTICS + ANTIHISTAMINES FOR SYSTEMIC USE | 2 (2.2%) |
| ranitidine (ARS=1) + loperamide (ARS=2) | DRUGS FOR ACID RELATED DISORDERS + ANTIDIARRHEALS, INTESTINAL ANTIINFLAMMATORY/ANTIINFECTIVE AGENTS | 1 (1.1%) |
| ranitidine (ARS=1) + loratadine (ARS=2) | DRUGS FOR ACID RELATED DISORDERS + ANTIHISTAMINES FOR SYSTEMIC USE | 2 (2.2%) |
| trimipramine (ARS=3) | PSYCHOANALEPTICS | 1 (1.1%) |
| chlorpromazine (ARS=3) + quetiapine (ARS=1) | PSYCHOLEPTICS + PSYCHOLEPTICS | 1 (1.1%) |
| hydroxyzine (ARS=3) + paroxetine (ARS=1) | PSYCHOLEPTICS + PSYCHOANALEPTICS | 2 (2.2%) |
| levodopa (ARS=1) + levodopa (ARS=1) + loratadine (ARS=2) | ANTI-PARKINSON DRUGS + ANTI-PARKINSON DRUGS + ANTIHISTAMINES FOR SYSTEMIC USE | 1 (1.1%) |
| loperamide (ARS=2) + loratadine (ARS=2) | ANTIDIARRHEALS, INTESTINAL ANTIINFLAMMATORY/ANTIINFECTIVE AGENTS + ANTIHISTAMINES FOR SYSTEMIC USE | 1 (1.1%) |
| metoclopramide (ARS=1) + hydroxyzine (ARS=3) | DRUGS FOR FUNCTIONAL GASTROINTESTINAL DISORDERS + PSYCHOLEPTICS | 2 (2.2%) |
| pramipexole (ARS=1) + hydroxyzine (ARS=3) | ANTI-PARKINSON DRUGS + PSYCHOLEPTICS | 1 (1.1%) |
| quetiapine (ARS=1) + trimipramine (ARS=3) | PSYCHOLEPTICS + PSYCHOANALEPTICS | 1 (1.1%) |
| ranitidine (ARS=1) + oxybutynin (ARS=3) | DRUGS FOR ACID RELATED DISORDERS + UROLOGICALS | 1 (1.1%) |
| amitriptyline (ARS=3) + loratadine (ARS=2) | PSYCHOANALEPTICS + ANTIHISTAMINES FOR SYSTEMIC USE | 1 (1.1%) |
| hydroxyzine (ARS=3) + loratadine (ARS=2) | PSYCHOLEPTICS + ANTIHISTAMINES FOR SYSTEMIC USE | 3 (3.3%) |
| loperamide (ARS=2) + hydroxyzine (ARS=3) | ANTIDIARRHEALS, INTESTINAL ANTIINFLAMMATORY/ANTIINFECTIVE AGENTS + PSYCHOLEPTICS | 3 (3.3%) |
| olanzapine (ARS=2) + hydroxyzine (ARS=3) | PSYCHOLEPTICS + PSYCHOLEPTICS | 1 (1.1%) |
| hydroxyzine (ARS=3) + amitriptyline (ARS=3) | PSYCHOLEPTICS + PSYCHOANALEPTICS | 1 (1.1%) |
| risperidone (ARS=1) + hydroxyzine (ARS=3) + loratadine (ARS=2) | PSYCHOLEPTICS + PSYCHOLEPTICS + ANTIHISTAMINES FOR SYSTEMIC USE | 1 (1.1%) |

ATC, Anatomical Therapeutic and Chemical

*The anticholinergic burden and CKD progression*

During the median [IQR] follow-up period of 5.0 [4.6-5.2] years, 647 patients initiated KRT (IR [95%CI]: 5.5 [5.1-6.0] per 100 person-years), 38 of them had a low or moderate anticholinergic burden (IR [95%CI]: 5.8 [3.9-7.6] per 100 person-years), and 25 had a high anticholinergic burden (IR [95%CI]: 8.0 [4.9-11.2] per 100 person-years) (**Table SM3**).

After adjustments for baseline sociodemographic factors, laboratory variables, comorbidities and drugs, a high anticholinergic burden was not significantly associated with a higher risk of CKD progression to KRT (HR [95%CI]: 1.43 [0.94-2.18]), relative to a null anticholinergic burden. A low or moderate anticholinergic burden was also not significantly associated with CKD progression to KRT (HR [95%CI]: 1.20 [0.86-1.68]), relative to a null anticholinergic burden (**Figure SM2**). The lack of significant associations might have been due to a loss of statistical power; however, the results observed for the ACB data were consistent with those observed for the ARS data. Similar results were observed when uACR was added to the model (**Table SM4)**.

## Table SM3: Incidence rate for CKD progression to KRT as a function of the anticholinergic burden (calculated using the ARS)

|  | **All**  N=3,009 | **Anticholinergic burden** (ARS) | | |
| --- | --- | --- | --- | --- |
|  |  | **Null**  N = 2,745 | **Low and moderate**  N = 174 | **High**  N = 90 |
| Person-years | 11689.0 | 10718.5 | 658.8 | 311.6 |
| Number of KRT initiations | 647 | 584 | 38 | 25 |
| Incidence rate per 100 person-years | 5.5 [5.1-6.0] | 5.4 [5.0-5.9] | 5.8 [3.9-7.6] | 8.0 [4.9-11.2] |

ARS, Anticholinergic Risk Scale; KRT, kidney replacement therapy


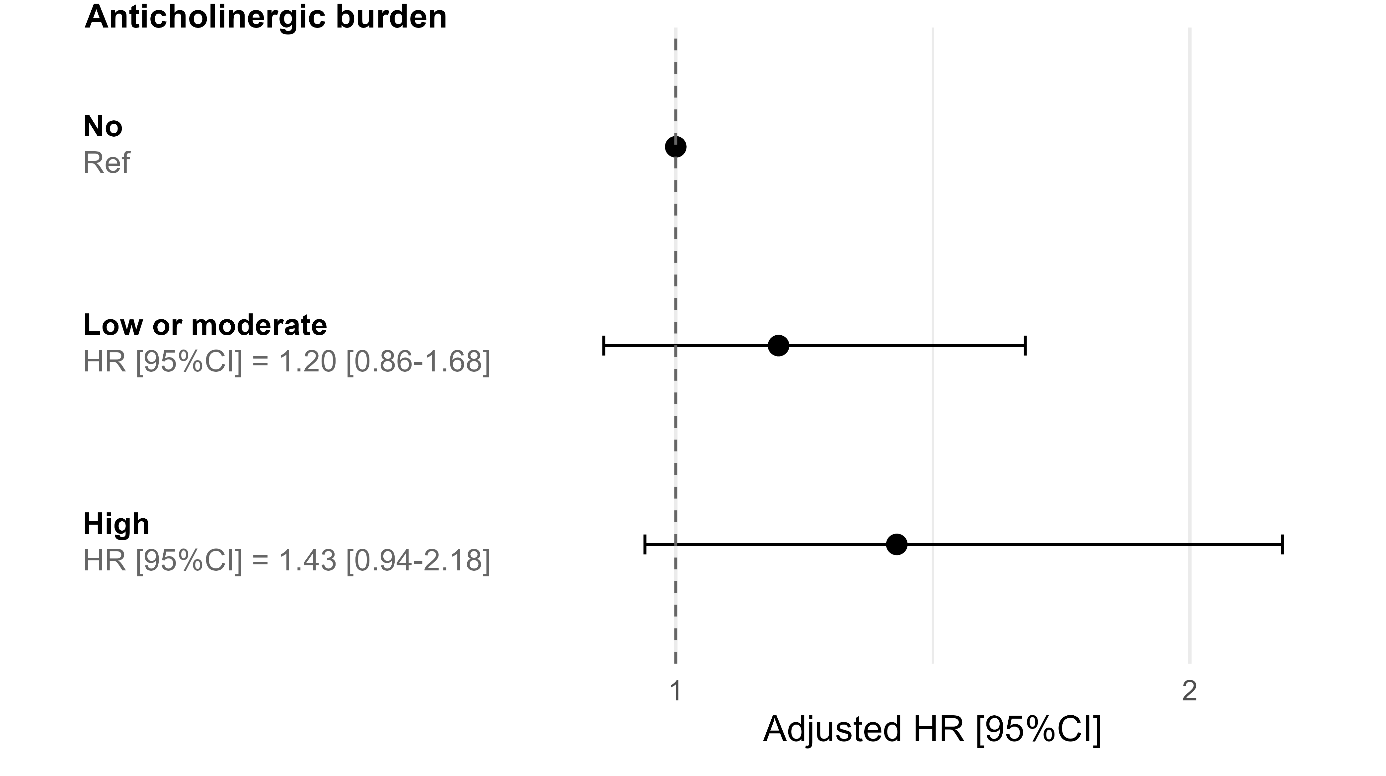


HR, hazard ratio; CI, confidence interval.

Hazard ratios were adjusted for age, sex, eGFR, systolic blood pressure (mmHg), history of cardiovascular disease, diabetes mellitus, dyslipidemia, autosomal dominant polycystic kidney disease, the number of prescription drugs taken daily, and compliance.

## Figure SM2: Adjusted hazard ratios for CKD progression to KRT and the anticholinergic burden (calculated using the ARS)

## Table SM4: Adjusted hazard ratios for CKD progression to KRT and the anticholinergic burden (calculated using the ARS), after the addition of urinary albumin-to-creatinine ratio (uACR) to the model

|  | **Sensitivity analysis** | **Sensitivity analysis + uACR** |
| --- | --- | --- |
| Null ARS | *Ref* | *Ref* |
| Low or moderate ARS | 1.20 [0.86-1.68] | 1.18 [0.84-1.65] |
| High ARS | 1.43 [0.94-2.18] | 1.40 [0.91-2.15] |

ARS, Anticholinergic risk scale; uACR, urinary albumin-to-creatinine ratio.

*Anticholinergic burden and death*

During the median [IQR] follow-up period of 5.0 [4.6-5.2] years, 405 patients died (IR [95%CI]: 3.5 [3.1-3.8] per 100 person-years): 28 of them had a low or moderate anticholinergic burden (IR [95%CI]: 4.3 [2.7-5.8] per 100 person-years), and 16 had a high anticholinergic burden (IR [95%CI]: 5.1 [2.6-7.6] per 100 person-years) (**Table SM5**).

After adjustment for sociodemographic factors, laboratory variables, comorbidities and drugs, our analysis did not reveal a significant association between the anticholinergic burden and death (HR [95%CI]: 1.12 [0.76-1.67] for a low and moderate anticholinergic burden and 0.88 [0.52-1.49] for a high anticholinergic burden, compared with a null anticholinergic burden) (**Figure SM3**). Despite a potential loss of statistical power, these results were consistent with those obtained with the ACB data. Similar results were observed when uACR was added to the model (**Table SM6)**.

## Table SM5: Incidence rate for death and the anticholinergic burden (calculated using the ARS)

|  | **All**  N=3,009 | **Anticholinergic burden** (ARS) | | |
| --- | --- | --- | --- | --- |
|  |  | **Null**  N = 2,745 | **Low and moderate**  N = 174 | **High**  N = 90 |
| Person-years | 11689.0 | 10718.5 | 658.8 | 311.6 |
| Number of death | 405 | 361 | 28 | 16 |
| Incidence rate for 100 person-years | 3.5 [3.1-3.8] | 3.4 [3.0-3.7] | 4.3 [2.7-5.8] | 5.1 [2.6-7.6] |

ARS: Anticholinergic Risk Scale


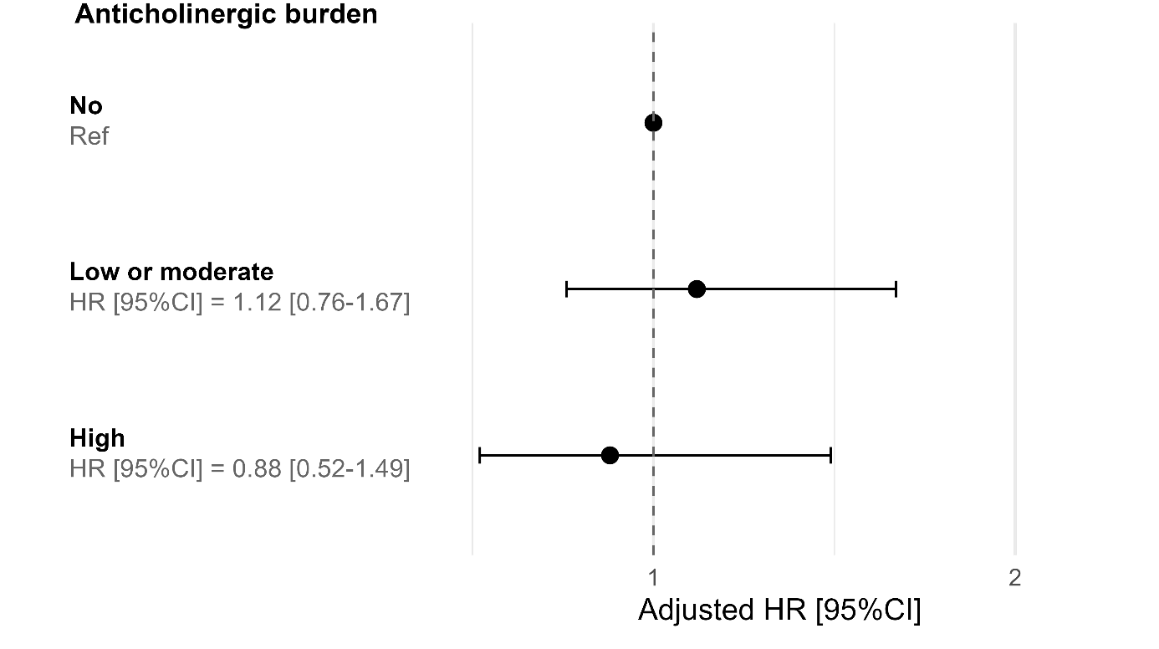


HR, hazard ratio; CI, confidence interval.

Note: Hazard ratios were adjusted for age, sex, educational level, smoking status, eGFR, urea, uric acid levels, body mass index, history of cardiovascular disease, systolic blood pressure, antihypertensive, lipid-modifying agents, compliance and the number of prescription drugs taken daily.

No interaction between sex and anticholinergic burden was detected.

## Figure SM3: Adjusted hazard ratios for death and the anticholinergic burden (calculated using the ARS)

## Table SM6: Adjusted hazard ratios for death and the anticholinergic burden (calculated using the ARS), after the addition of the urinary albumin-to-creatinine ratio (uACR) to the model

|  | **Sensitivity analysis** | **Sensitivity analysis + uACR** |
| --- | --- | --- |
| Null ARS | *Ref* | *Ref* |
| Low or moderate ARS | 1.12 [0.76-1.67] | 0.89 [0.44-1.78] |
| High ARS | 0.88 [0.52-1.49] | 0.58 [0.21-1.62] |

ARS, Anticholinergic risk burden; uACR, urinary albumin-to-creatinine ratio.

STROBE Statement—Checklist of items that should be included in reports of ***cohort studies***

|  | Item No | Recommendation | Page No |
| --- | --- | --- | --- |
| **Title and abstract** | 1 | (*a*) Indicate the study’s design with a commonly used term in the title or the abstract | 2 |
|  |  | (*b*) Provide in the abstract an informative and balanced summary of what was done and what was found | 2 |
| Introduction | | |  |
| Background/rationale | 2 | Explain the scientific background and rationale for the investigation being reported | 3-4 |
| Objectives | 3 | State specific objectives, including any prespecified hypotheses | 4 |
| Methods | | |  |
| Study design | 4 | Present key elements of study design early in the paper | 4 |
| Setting | 5 | Describe the setting, locations, and relevant dates, including periods of recruitment, exposure, follow-up, and data collection | 4-5 |
| Participants | 6 | (*a*) Give the eligibility criteria, and the sources and methods of selection of participants. Describe methods of follow-up | 4 |
|  |  | (*b*) For matched studies, give matching criteria and number of exposed and unexposed |  |
| Variables | 7 | Clearly define all outcomes, exposures, predictors, potential confounders, and effect modifiers. Give diagnostic criteria, if applicable | 5-7 |
| Data sources/ measurement | 8* | For each variable of interest, give sources of data and details of methods of assessment (measurement). Describe comparability of assessment methods if there is more than one group | 4-7 |
| Bias | 9 | Describe any efforts to address potential sources of bias | 5-8 |
| Study size | 10 | Explain how the study size was arrived at |  |
| Quantitative variables | 11 | Explain how quantitative variables were handled in the analyses. If applicable, describe which groupings were chosen and why | 4-7 + Table S1 |
| Statistical methods | 12 | (*a*) Describe all statistical methods, including those used to control for confounding | 6-8 |
|  |  | (*b*) Describe any methods used to examine subgroups and interactions | 7-8 |
|  |  | (*c*) Explain how missing data were addressed | 8 |
|  |  | (*d*) If applicable, explain how loss to follow-up was addressed |  |
|  |  | (*e*) Describe any sensitivity analyses | 8 |
| Results | | |  |
| Participants | 13* | (a) Report numbers of individuals at each stage of study—eg numbers potentially eligible, examined for eligibility, confirmed eligible, included in the study, completing follow-up, and analysed | 9 |
|  |  | (b) Give reasons for non-participation at each stage | 9 |
|  |  | (c) Consider use of a flow diagram |  |
| Descriptive data | 14* | (a) Give characteristics of study participants (eg demographic, clinical, social) and information on exposures and potential confounders | 9 + Table 1 |
|  |  | (b) Indicate number of participants with missing data for each variable of interest | Table 1 |
|  |  | (c) Summarise follow-up time (eg, average and total amount) | 9-10 |
| Outcome data | 15* | Report numbers of outcome events or summary measures over time | 9-10 |
| Main results | 16 | (*a*) Give unadjusted estimates and, if applicable, confounder-adjusted estimates and their precision (eg, 95% confidence interval). Make clear which confounders were adjusted for and why they were included | 9-10 |
|  |  | (*b*) Report category boundaries when continuous variables were categorized | Table 1 + Table S1 |
|  |  | (*c*) If relevant, consider translating estimates of relative risk into absolute risk for a meaningful time period |  |
| Other analyses | 17 | Report other analyses done—eg analyses of subgroups and interactions, and sensitivity analyses | 10-11 + supplementary materials |
| Discussion | | |  |
| Key results | 18 | Summarise key results with reference to study objectives | 12 |
| Limitations | 19 | Discuss limitations of the study, taking into account sources of potential bias or imprecision. Discuss both direction and magnitude of any potential bias | 12-15 |
| Interpretation | 20 | Give a cautious overall interpretation of results considering objectives, limitations, multiplicity of analyses, results from similar studies, and other relevant evidence | 12-15 |
| Generalisability | 21 | Discuss the generalisability (external validity) of the study results | 12-15 |
| Other information | | |  |
| Funding | 22 | Give the source of funding and the role of the funders for the present study and, if applicable, for the original study on which the present article is based | 18 |

*Give information separately for exposed and unexposed groups.

**Note:** An Explanation and Elaboration article discusses each checklist item and gives methodological background and published examples of transparent reporting. The STROBE checklist is best used in conjunction with this article (freely available on the Web sites of PLoS Medicine at http://www.plosmedicine.org/, Annals of Internal Medicine at http://www.annals.org/, and Epidemiology at http://www.epidem.com/). Information on the STROBE Initiative is available at http://www.strobe-statement.org.
